# A Cu_9_S_5_ nanoparticle-based CpG delivery system for synergistic photothermal-, photodynamic- and immunotherapy

**DOI:** 10.1038/s42003-020-1070-6

**Published:** 2020-07-03

**Authors:** Lulu Zhou, Lv Chen, Xiaochun Hu, Yonglin Lu, Wenjie Liu, Yanting Sun, Tianming Yao, Chunyan Dong, Shuo Shi

**Affiliations:** Shanghai Key Laboratory of Chemical Assessment and Sustainability, School of Chemical Science and Engineering, Breast Cancer Center, Shanghai East Hospital, Tongji University, 200092 Shanghai, P. R. China

**Keywords:** Drug delivery, Biomedical materials, Cancer immunotherapy, Cancer therapy

## Abstract

Despite its great potential in cancer therapy, phototherapy, including photothermal therapy (PTT) and photodynamic therapy (PDT), often cause metastasis of tumors. Immunotherapy has revolutionized the cancer treatment owing to the capability of activating immune system to eliminate tumors. However, the integration of phototherapy and immunotherapy in a single nanoagent for cancer therapy is still a challenging task. Here, we fabricated (Cu_9_S_5_@mSiO_2_-PpIX@MnO_2_@CpG (CSPM@CpG)) as a synergistic therapeutic model for phototherapy enhanced immunotherapy. The intracellular uptake of cytosine-phosphate-guanine (CpG) promoted the infiltration of cytotoxic T lymphocytes (CTLs) in tumor tissue, further stimulating the production of interferon gamma (IFN-γ) and remarkably elevating the immune response level. Excellent anti-tumor effects have been achieved by synergistic PTT/PDT/immunotherapy. The metastasis of tumors was effectively inhibited by the immune response of CpG. Thus, our proposed work provides a strategy to combine phototherapy with immunotherapy to enhance the therapeutic efficiency and further inhibit metastasis of tumors.

## Abstract (web summary)

CpG oligonucleotides can stimulate an anticancer immune response. Zhou et al. now develop nanoparticles that can deliver CpG, which promotes cancer cell death after photothermal and photodynamic therapy in vitro and in vivo, concomitant with an immunological response.

## Introduction

The traditional and main strategies for cancer treatment, such as surgical resection, chemotherapy, and radiotherapy, always accompany with systemic side effects including high metastasis rate or toxicity to the immune system and normal tissues, so it is considerably urgent to develop accurate and efficient tumor-therapeutic modalities^1,2^. As a novel and minimally invasive alternative to traditional therapeutic methodology, phototherapy, including photothermal therapy (PTT) and photodynamic therapy (PDT), has attracted widespread interest owing to its efficient cancer destruction ability, fewer side effects and minimal invasiveness^3–7^.

The process of PTT involves that photothermal agents exposed to near infrared (NIR) light can efficiently convert the optical energy into thermal energy and ablate target tumors by hyperthermia^8^. Recently, copper sulfide compounds have been receiving a great deal of interest due to their strong NIR absorption derived from p-type carriers in vacancy-doped nanocrystals^9,10^, low cost, easy synthesis, and high thermal conversion efficiency^11^. In parallel, the photosensitizer during PDT can convert surrounding oxygen molecules into cytotoxic reactive oxygen species (ROS) under light irradiation, especially singlet oxygen (^1^O_2_), causing irreversibly protein or DNA damage, subsequently triggering apoptosis and necrosis of tumor cells^12,13^. In general, the mechanism of PDT fall into two categories: type I PDT, producing superoxide (O_2_^∙−^) or hydroperoxyl radicals (HO_2_^∙^), and type II PDT, yielding ^1^O_2_, and the predominant PDT mechanism is type II^14^. Additionally, the therapeutic efficacy of conventional type II PDT largely depends on the amount of oxygen in the tumor^15^. But hypoxia is a distinct feature of most solid tumors, deriving from the imbalance of oxygen supply and consumption. The consumption of oxygen is caused by uncontrolled proliferation of cancer cells. In addition, the shutdown effects of vascular mediated by PDT would further aggravate hypoxia and in turn limit PDT efficiency^16,17^. To solve this problem, catalysts (catalase and MnO_2_)^18–20^ were used to improve the local O_2_ partial pressure in tumors through the decomposition of endogenous H_2_O_2_.

Both PTT and PDT have emerged as visible treatment options for malignant tumors, but their inherent shortcomings, especially metastasis of tumors after phototherapy, significantly hinder their extensive application in cancer treatment^21^. Fortunately, immunotherapy, especially the blockade of immune checkpoints, trains the host immune system to eliminate tumors by prompting surveillance and defense and has revolutionized the cancer treatment^22–24^. The programmed cell death-1 (PD-1) and its major ligand, programmed cell death-ligand 1 (PD-L1) signaling pathway, are a critical immune checkpoint, and its inhibition using monoclonal antibodies could reverse T cell dysfunction or exhaustion, which can provide a systemic therapy, including local tumor and metastatic lesion^25,26^. Among immunoadjuvants, unmethylated CpG oligonucleotides are specifically identified by Toll-like receptor 9 (TLR9) in plasmacytoid dendritic cells^26–30^ and thus stimulate an immune response to produce helper T cell 1 (Th1) and a pro-inflammatory cytokine (such as tumor necrosis factor alpha, TNF-α; Interluekin 12, IL-12)^21^. IL-12, as one of most effective stimulator of CTLs, could promote maturity of CTLs and infiltration in tumor site. At the same time, activated CTLs could secrete IFN-γ to kill tumor cells. However, tumor cells could induce CTLs exhaustion through PD-L1 signaling pathway for immune evasion, which could be effectively blockaded by aPD-L1 in turn^31,32^. So PD-1/PD-L1 checkpoint-blockade immunotherapy held a great expectation in cancer treatments. However, several challenges in clinical application of CpG still exist, such as being degraded by nucleases easily, hardness to cross the cell membrane^33^, unfavorable pharmacokinetic, and biodistribution characteristics^34^. So, it is critical to develop an efficient platform for CpG delivery and further protecting CpG from degradation. Up to now, liposomes^35^, carbon nanotubes^36^, hollow CuS nanoparticles^37^, and MoS_2_ nanosheets^38^ have been reported owing to their capacity to enhance CpG delivery and activate immunological effects to destroy the remaining cancer cells, thus further preventing metastasis^33,38–41^.

In light of the above considerations, multifunctional tumor-therapeutic platform, CSPM@CpG nanocomposite, was constructed by coating mesoporous silica shell and MnO_2_ shell successively on the surface of Cu_9_S_5_ nanocrystals, followed by adsorption of immunoadjuvant (CpG) for synergistic phototherapy and immunotherapy. The internalized CSPM@CpG nanocomposite could efficiently produce ROS and lots of heat under 650 nm laser illumination and 808 nm NIR laser irradiation, respectively, leading to the death of cancer cells. Besides, we found that this nanocomposite could promote the uptake of CpG and enhance the production of IL-12, TNF-α, and IFN-γ. Both in vitro and in vivo studies demonstrated that this multifunctional platform showed significantly enhanced tumor inhibition induced by phototherapy improved immunotherapy.

## Results

### Fabrication and characterizations of CSPM@CpG nanocomposite

**Fig. 1:**
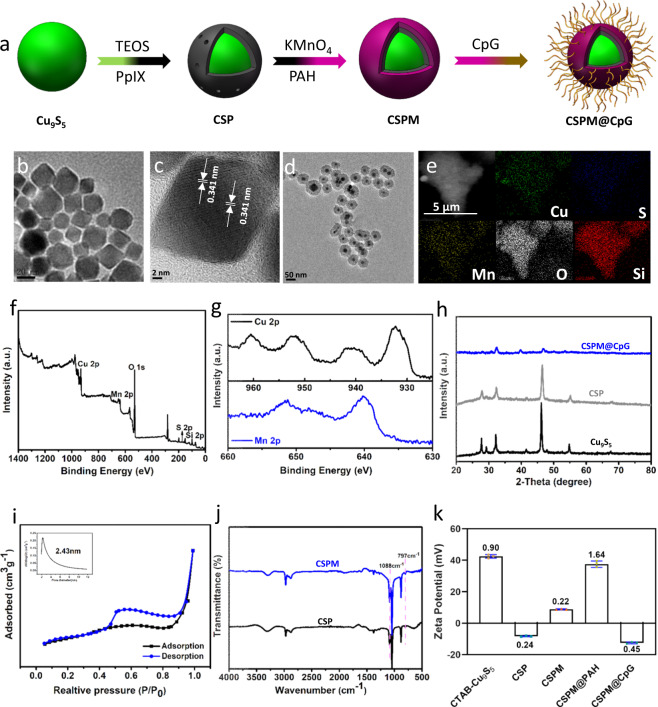
Fabrication and characterizations of CSPM@CpG. **a** A schematic illustration for the step-by-step synthesis of CSPM@CpG nanocomposite. **b**, **c** TEM image (**b**) and HRTEM image (**c**) of Cu_9_S_5_. **d** TEM image of CSP. **e** The overall SEM-EDX mapping images of Cu, S, Si, O, and Mn elements. **f**, **g** XPS spectrum (**f**) of CSPM@CpG and spectrum (**g**) of Cu2p, Mn2p. **h** XRD patterns of Cu_9_S_5_, CSP, and CSPM@CpG. **i** N_2_ adsorption/desorption isotherms (inset is the corresponding pre-size distribution) of CSPM@CpG. **j** FT-IR spectra of the CSP and CSPM. **k** Zeta-Potential of sample at different synthetic steps. Data are presented as means ± standard deviation (s.d.) (*n* = 3).

The rational design and preparation of CSPM@CpG are illustrated in Fig. 1a. First of all, the core of Cu_9_S_5_ nanocrystals were synthesized by a thermal decomposition^42^. Then these nanocrystals were transferred into an aqueous phase using cetyltrimethylammonium bromide (CTAB). Subsequently, the as-synthesized Cu_9_S_5_-CTAB was coated with a mesoporous silica (mSiO_2_) shell which PpIX was covalently conjugated on, resulting in the formation of Cu_9_S_5_@mSiO_2_-PpIX (CSP) core–shell composite nanoparticles. Thereafter, a MnO_2_ shell directly grown on the silica layer through the reduction of KMnO_4_ on the silica layer^43^ was used for tumor microenvironment-responsive generation of O_2_ to relieve tumor hypoxia, thereby achieving an enhanced photodynamic therapy. Lastly, CpG was successfully decorated on the surface of Cu_9_S_5_@mSiO_2_-PpIX@MnO_2_ (CSPM) to obtain CSPM@CpG through electrostatic interaction for later immunotherapy.

Transmission electron microscopy (TEM) image shows that Cu_9_S_5_ nanocrystals possess a uniform morphology with a mean size of ∼20 nm (Fig. 1b). And the high crystallinity of Cu_9_S_5_ is evidenced by high-resolution TEM (HRTEM) image (Fig. 1c). It can be clearly observed that a silica layer on CSP owing to the different electron penetrability between Cu_9_S_5_ and SiO_2_ shell (Fig. 1d). TEM image also indicates CSP nanoparticles were well-dispersed with average diameter of 47.6 nm. Further, the reduction of KMnO_4_ and the adsorption of CpG on the surface of CSP yields CSPM@CpG nanoparticles with an unaltered core (Supplementary Fig. 1a). Electron diffraction spectroscopy (EDS) spectrum indicates that CSPM@CpG consisted of the elements of Cu, S, Si, and Mn (Supplementary Fig. 1b). In addition, elemental mapping images showed that the elements mentioned above are simultaneously distributed in CSPM@CpG NPs, further confirming the successful formation of the as-expected product (Fig. 1e). Moreover, X-ray photoelectron spectroscopy (XPS) was then measured to verify above results and determine the valence of copper and manganese. The survey spectrum demonstrated that the sample is composed of Cu, S, Si, and Mn atoms (Fig. 1f), which is consistent with Supplementary Fig. 1b. In particularly, Cu 2p peaks include Cu 2p_3/2_ at 932.2 eV with the satellite peak at 941.4 eV and the Cu 2p_1/2_ at 952.7 eV with the satellite peak at 960.5 eV, which are in accordance with the Cu 2p orbital of Cu(II)^44^. The high-resolution XPS showed that Mn 2p peaks are centered at 651.2 and 640.2 eV, assigned to Mn 2p_1/2_ and Mn 2p_3/2_ peak, respectively (Fig. 1g), and this reveals that the main oxidation state of Mn is +4^45^. The size distributions of Cu_9_S_5_, CSP, and CSPM@CpG were obtained by dynamic light scattering (DLS) (Supplementary Fig. 2). To test the stability of CSPM@CpG, the nanocomposite was dispersed in PBS buffer solutions at different pH values at 37 °C or 43 °C within 12 h. And there were negligible changes about the sizes and zeta-potential of CSPM@CpG in different solutions (Supplementary Fig. 3). Additionally, no obvious changes about the CpG concentration and zeta-potential of CSPM@CpG before and after laser irradiation were observed (Supplementary Table 1). The high stability of CSPM@CpG suggests their potential for in vitro and in vivo therapeutic applications.

X-ray powder diffraction (XRD) pattern of Cu_9_S_5_ core nanoparticles is consistent with that of standard Cu_9_S_5_ phase (JCPDS card No: 47-1748), with several well-defined characteristic peaks such as (0, 0, 15), (1, 0, 10), and (0, 1, 20) (Supplementary Fig. 4). And all the peak positions of as-prepared CSP and CSPM@CpG can be directly fitted with the standard of Cu_9_S_5_ (Fig. 1h). In comparison with CSP, no diffraction peaks of MnO_2_ and meanwhile the weaken peak intensity were observed in the XRD pattern of MnO_2_ shell-coated CSPM, implying the amorphous nature of MnO_2_ layer. The N_2_ adsorption/desorption isotherms of CSPM@CpG can be classified as type IV, which is the characteristic of typical mesoporous materials (Fig. 1i). The average pore diameter was calculated to be 2.43 nm (inset of Fig. 1i) using Barrett–Joyner–Halenda (BJH) model. In addition, Brunauer–Emmett–Teller (BET) surface area and BJH pore volume are calculated to be 206.50 m² g^−1^ and 0.044 cm³ g^−1^, respectively. Fourier-transform infrared (FT-IR) spectra of the samples in every step were conducted to confirm the functional groups on the sample surface. After coating with silica shell (the black curve), typical peaks at 797 cm^−1^ and 1088 cm^−1^ are assigned to the Si–O–Si deformation vibrations and asymmetric stretching, respectively (Fig. 1j). CSPM has a similar FT-IR spectrum to CSP because of the similar functional groups (the blue curve). Fig.1k exhibits the measured zeta potentials of CSP, CSPM, CSPM@PAH, and CSPM@CpG. It can be clearly seen that the CTAB-stabilized Cu_9_S_5_ nanocrystals possess the positive charge (+43.5 mV) because of the adsorption of cationic surfactant (CTAB). However, CTAB used as a template has been found to be cytotoxic, so it is critical to remove it before biological applications^41^. After extraction by the mild acetone thrice, the zeta potential of CSP core–shell nanoparticles changed from positive charge to negative charge (−8.49 mV), indicating the template was gradually removed from the mesoporous silica shells. Then the assembly of the MnO_2_ shell on CSP nanoparticles contributes to an increase in the zeta potential to +8.64 mV. After being adsorbed with cationic PAH, the zeta potential value of CSPM@PAH increase to +39.4 mV. The obtained CSPM@CpG possesses a zeta potential of −12 mV, indicating the successful anchor of CpG on the surface of CSPM@PAH, owing to the negative charge of CpG.

**Fig. 2:**
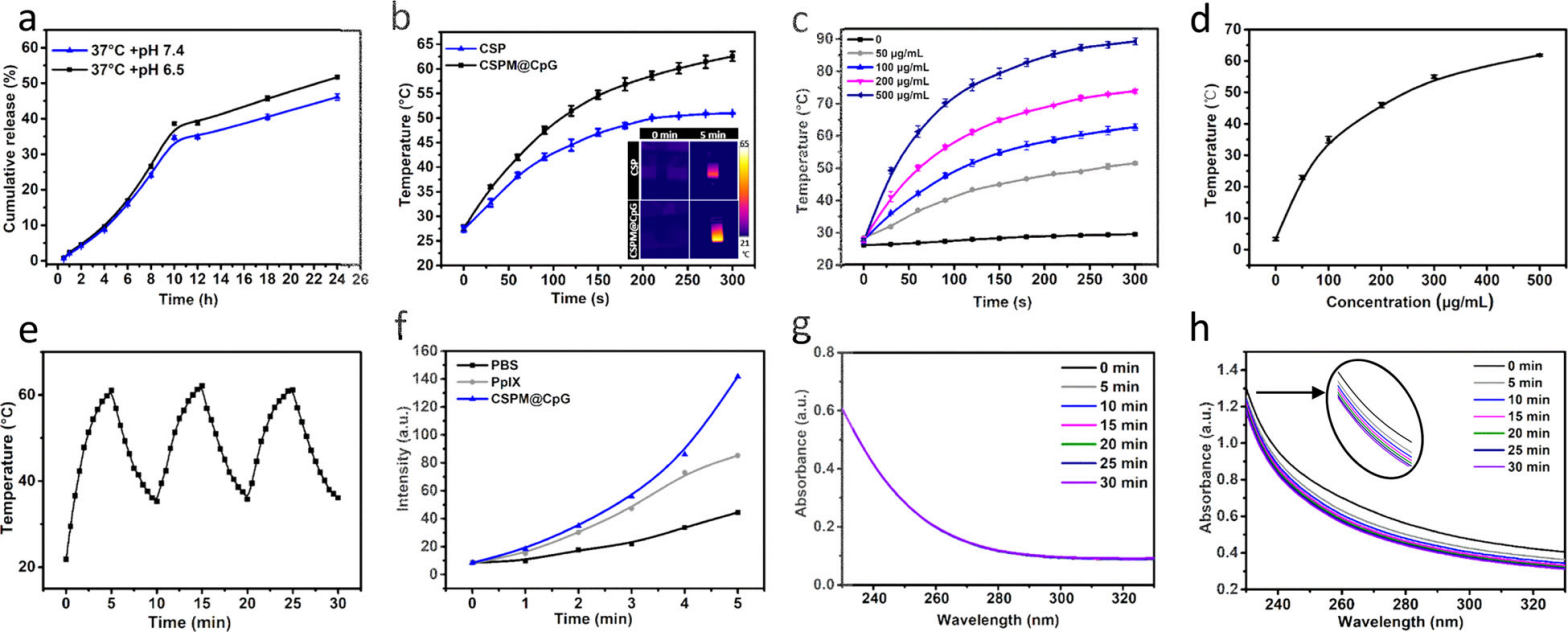
Drug release behavior, phototherapy, and catalase-like performance of CSPM@CpG. **a** Percentage of cumulative released CpG from CSPM@CpG at different pH values (7.4 and 6.5). **b** Comparison of temperature increases CSP and CSPM@CpG (100 μg mL^−1^) under 808 nm irradiation at 2 W cm^−2^. **c** Temperature change of CSPM@CpG under different concentrations. **d** Plot of the temperature elevation over a period of 300 s versus the concentration of CSPM@CpG. **e** Temperature variation curves of a CSPM@CpG solution (100 μg mL^−1^) under 3 heating/cooling cycles (808 nm laser, 2 W cm^−2^). **f** ROS detection of CSPM@CpG nanocomposite (100 μg mL^−1^) with DCFH under 650 nm laser irradiation at 1 W cm^−2^ for 5 min (monitored once per minute). **g**, **h** UV-Vis spectra of remainder H_2_O_2_ were recorded after reaction with (**g**) PBS and (**h**) CSPM@CpG for different times in pH 7.4, respectively. Data are presented as means ± standard deviation (s.d.) (*n* = 3).

Based on UV-Vis adsorption spectra, CSP nanoparticles showed a new absorption peak attributed to the PpIX unit compared with the absorption of Cu_9_S_5_@mSiO_2_ nanocrystals, indicating the successful link of PpIX by covalently bound onto the nanocrystals (Supplementary Fig. 5a). After CpG was loaded on the surface of MnO_2_, UV-Vis spectrum of CSPM@CpG showed the characteristics of both CSP NPs and CpG (Supplementary Fig. 5b), but the intensity of PpIX was reduced when compared with CSP, ascribing to the absorbability of black-colored MnO_2_^43^. According to the standard absorption curve of PpIX on the basis of Lambert–Beer law (Supplementary Fig. 5c, d), the loading efficiency of PpIX was determined to be 4.73% and the encapsulating efficiency was 80.3%. In addition, the loading efficiency was calculated to be ~14.8% for CpG based on Nanodrop. Then the release behavior of CpG was studied in solutions at different pH values at 37 °C or 43 °C (Fig. 2a and Supplementary Fig. 5e). Notably, the CpG release was slightly faster under acidic conditions (pH = 6.5) compared with that under the physiological condition (pH = 7.4) at 37 °C or 43 °C after 24 h, demonstrating that CSPM@CpG nanocomposite possessed pH-responsive release behavior for CpG.

### Photothermal, photodynamic, and catalase-like performance with CSPM@CpG

The strong adsorption in the NIR region of CSP (Supplementary Fig. 6a) endows CSPM@CpG nanocomposite with a photothermal property. It was noteworthy that CSPM@CpG exhibited more excellent photothermal effect in comparison with CSP under the same conditions and the temperature of CSPM@CpG was 15 °C higher than that of CSP (100 μg mL^−1^) after 5 min of 808 nm NIR irradiation (Fig. 2b). From the infrared thermal images (inset of Fig. 2b), it could be also clearly observed that CSPM@CpG showed higher temperature change than CSP. We speculated that surface plasmon coupling effect generated by CSPM@CpG nanocomposite highly improved local electromagnetic field, thus extending beyond the resonant plasmon excitation energy and resulting in an elevated heat-generating ability^46^. Under the irradiation of 808 nm laser (2 W cm^−2^), the temperature change showed a concentration and irradiation time-dependent pattern, which meant the temperature increase accordingly with the increase of concentration and irradiation time (Fig. 2c and Supplementary Fig. 6b). After 5 min of irradiation, the temperature of all CSPM@CpG solutions increased obviously as high as ~80 °C, indicating the great potential of CSPM@CpG to be a photothermal agent (Fig. 2d). Particularly, at a concentration of 100 μg mL^−1^, the temperature changed as high as 39.3 °C, increasing from 21.8 °C to 61.1 °C, whereas the temperature of PBS just increased to 29.1 °C under the same irradiation conditions. Subsequently, the photostability experiment of CSPM@CpG solution (100 μg mL^−1^) was conducted for successive three on/off laser cycles, during which 5 min of 808 nm irradiation (laser on) and a natural cooling process without NIR laser irradiation for 5 min (laser off) were applied. As expected, the repeated heating/cooling cycles had no significant impact on the temperature change (Fig. 2e), which demonstrated the great photostability and reproducibility of CSPM@CpG nanocomposite. These results successfully indicated that as obtained CSPM@CpG nanocomposite could serve as a good photothermal agent.

To further investigate the photodynamic effect of CSPM@CpG nanocomposite, a solution containing 100 μg mL^−1^ nanocomposite and 2 mM 2′, 7′-Dichlorofluorescein (DCFH) was exposed to NIR irradiation for 5 min (650 nm, 1 W cm^−2^). DCFH was applied as a fluorescent probe to evaluate the production of ROS^47^. In the presence of CSPM@CpG, the fluorescence of DCF increased obviously along with the increase of time, indicating the superior ^1^O_2_ generation ability of nanocomposite (Fig. 2f). Additionally, to identify whether the heat during PTT would affect the generation of ^1^O_2_, the ^1^O_2_ production of CSPM@CpG under 650 nm, 808 nm, and 650 nm + 808 nm laser irradiation was studied (Supplementary Fig. 6c). The fluorescence intensity after dual laser irradiation was enhanced dramatically compared with that after single laser irradiation, which was due to the combined effect originating from O_2_^∙−^ or HO_2_^∙^ generated by Cu_9_S_5_^48^ and ^1^O_2_ produced by PpIX. So CSPM@CpG nanocomposite can serve as an efficient photosensitizer for PDT application in cancer treatment.

It is well known that there are excessive amounts of H_2_O_2_ produced by malignant cancer cells. As hydrogen peroxide can be decomposed by MnO_2_ into oxygen, and then the catalase capacity of CSPM@CpG NPs was estimated by evaluating the consumption of H_2_O_2_ (10 mM) through the decomposition. The obvious decrease of H_2_O_2_ concentration in the sample containing CSPM@CpG NPs (100 μg mL^−1^) under physiological pH condition (pH 7.4) was recorded by UV-Vis spectra (Fig. 2g, h). By contrast, there were negligible changes of the control group (only containing H_2_O_2_). In addition, as the catalytic ability of catalysts was affected by pH, the catalase-like capacity of CSPM@CpG was further measured at pH 6.5, which was the characteristic pH of the tumor microenvironment owing to the exuberant metabolism of cancer cells. As expected, CSPM@CpG also showed high catalytic activity at pH 6.5 (Supplementary Fig. 6d, e). All of these results indicated that CSPM@CpG possessed a catalase-like property at both pH 7.4 and pH 6.5 for inducing the decomposition of H_2_O_2_ to produce O_2_.

### Cellular experiments

**Fig. 3:**
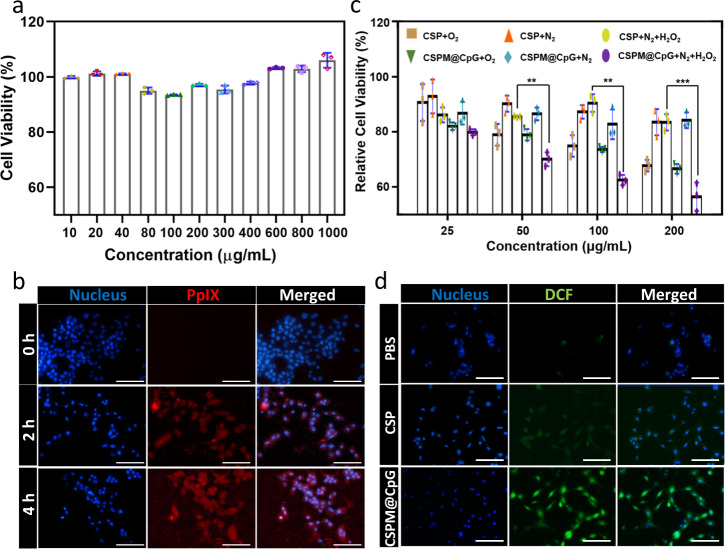
In vitro experiments with CSPM@CpG. **a** The viabilities of 4T1 cells incubated with different concentrations CSPM@CpG (from 10 to 1000 μg mL^−1^) for 24 h. **b** CLSM images of 4T1 cells incubated with CSPM@CpG for different time periods. **c** In vitro PDT treatment of 4T1 cells by CSP or CSPM@CpG with or without addition of H_2_O_2_ under 650 nm light irradiation in O_2_ or N_2_ atmospheres. **d** Detection of intracellular ROS in 4T1 cells incubated with CSP and CSPM@CpG upon the addition of H_2_O_2_ under 650 nm irradiation in N_2_ atmospheres. Scale bar: 100 μm. Data are presented as means ± standard deviation (s.d.; *n* = 3). *p* values **<0.01, ***<0.001.

Cellular experiments were further conducted to determine the effectiveness of our nanocomposite to serve as agents for PTT/PDT/immunotherapy. First, to make sure CSPM@CpG possess high biocompatibility and high phagocytosis for the further treatment, cell viability and cellular uptake efficiency of CSPM@CpG were evaluated on 4T1 cells. It was found that CSPM@CpG showed no obviously adverse effect on cells even at high concentrations up to 1000 μg mL^−1^, which reflected CSPM@CpG possessed an excellent biocompatibility for biological application (Fig. 3a). Such high biosafety was owing to the low toxicity of the precursor of the composites^42^. Then the cellular uptake efficiency of CSPM@CpG was assessed by confocal laser scanning microscope (CLSM) and Cu^2+^ content per well after incubation with cells for 4 h through ICP measurement. The results revealed that with prolonging of incubation time, an enhanced red fluorescence accumulated in the cells was observed (Fig. 3b and Supplementary Fig. 7). Meanwhile, with the increase of concentration, the more Cu^2+^ content taken by 4T1 cells was observed (Supplementary Fig. 8). It was indicated that CSPM@CpG was effectively internalized in 4T1 cells.

**Fig. 4:**
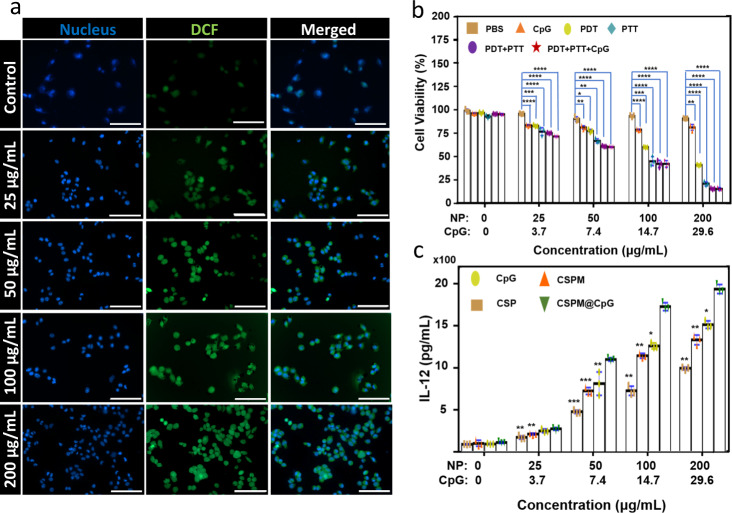
In vitro experiments with CSPM@CpG. **a** Detection of intracellular ROS in 4T1 cells of PBS group and different concentrations of CSPM@CpG groups under 650 nm laser irradiation at 1 W cm^−2^ for 5 min. Scale bar: 100 μm. **b** Cell viability of 4T1 cells incubated with different concentrations of CSPM@CpG NPs under PDT treatment (650 nm, 1 W cm^−2^, 5 min), PTT treatment (808 nm, 2 W cm^−2^, 5 min), and combined PDT treatment and PTT treatment. **c** The secretion of IL-12 from DC 2.4 cells stimulated with different treatments. The concentration of CpG was consistent with that loaded in the CSMP@CpG. Data are presented as means ± standard deviation (s.d.; *n* = 3). *p* values *<0.05, **<0.01, ***<0.001, ****<0.0001.

To evaluate the PDT efficacy of MnO_2_-coated CSP nanoparticles, 4T1 cells were incubated with CSP and CSPM@CpG with or without exogenous 100 µM H_2_O_2_, then irradiated by 650 nm laser for 30 min (1 W cm^−2^) in either O_2_ or N_2_ environment. After incubation for another 24 h with fresh cell medium, the cell viability was used to determine the PDT efficacy (Fig. 3c). Although the PDT-induced cell killing under the nitrogen atmosphere was found to be much less effective, the PDT efficacy of CSPM@CpG was dramatically intensified in the case of exogenous H_2_O_2_ adding but not for CSP NPs without MnO_2_ coating. Subsequently, the level of intracellular ROS induced by CSPM@CpG under irradiation was also investigated by CLSM using 2′,7′-dichlorofluorescein diacetate (DCFH-DA) as fluorescent probe. In comparison to CSP, an enhanced fluorescence of CSPM@CpG under hypoxic environment with the exogenous H_2_O_2_ was observed (Fig. 3d), likely due to the additional supply of oxygen by MnO_2_-trigged decomposition of H_2_O_2_. Additionally, much stronger fluorescence was observed with the increasing concentration of CSPM@CpG in O_2_ atmosphere (Fig. 4a), which could be ascribed to the reaction of DCFH-DA with the larger amounts of ROS generated. All of the above results demonstrated that CSPM@CpG would offer a chance for enhanced-PDT to attain a more effective anti-tumor therapy.

The in vitro anti-tumor efficiency was determined by the viability of 4T1 cells incubated with different composites under light irradiation (Fig. 4b). Under 650 nm laser (1 W cm^−2^) and 808 nm laser (2 W cm^−2^) simultaneous irradiation for 5 min, the cell viability treated with CSPM NPs was significantly decreased, showing a dose-dependent manner. When the concentration of CSPM NPs reached 200 μg mL^−1^, the cell viability declined to 15.3%. It was also found that the combined treatment induced a dramatically higher cytotoxicity compared to those after treatment by either PDT or PTT alone. Take the concentration of 100 μg mL^−1^ as an example, the viability of 4T1 cells treated with CSPM nanocomposite for PDT, PTT, and combined PDT and PTT was 77.6, 59.2, and 41.6%, respectively. And the groups treated with PDT, PTT, and immunotherapy showed negligible change in cell viability (41.60% at 100 μg mL^−1^) compared to those treated with combined PDT and PTT therapy (41.62% at 100 μg mL^−1^). The results could be ascribed to the incapability of CpG to induce the apoptosis of 4T1 cells without invoking the antigen presenting cells in vitro^21^. The combination index (CI) of PTT-PDT-immunotherapy was calculated to be 0.9, according to the Chou-Talalay method^49^. The anti-tumor efficiency of CSPM@CpG in vitro was also evaluated by cell apoptosis assay with Annexin V-FITC Apoptosis Detection Kit (Supplementary Fig. 9). The involvement of dual staining, green emission from Annexin V-FITC bound to phosphatidyl serine and red emission from DNA binding PI dye to identify the cellular apoptotic (early or late) or necrotic pathways. During the early stage of apoptosis, cells bind to Annexin V-FITC and change their phospholipid asymmetry to show green fluorescence while necrotic cells bind only with PI. In late apoptosis, the cells lose their membrane integrity and display dual positive emission. Under combined 650 nm and 808 nm irradiation for 5 min, the ratio of dead cells and live cells for CSPM@CpG is 28.8%, much higher than 0% for single 650 nm irradiation (22.3% apoptotic cells) and 22.6% for 808 nm irradiation alone. These results were consistent with the cytotoxicity results. In addition, as observed in Supplementary Fig. 9, the apoptotic cells at an early stage with PDT treatment could be transformed to dead cells with the treatment of PTT, which demonstrate that PTT has an excellent anti-cancer effect.

Next, the in vitro immune-stimulatory activity of CSPM@CpG was evaluated. CpG, CSP, CSPM, and CSPM@CpG were incubated with DC 2.4 cells, respectively. CpG could be taken by DC cells (infiltration of phagocytosis by this population was shown in Supplementary Fig. 10). The secretion of cytokines, IL-12, as an indicator of immune response, was the most important stimulator of CTL and was measured by ELISA. For CSP and CSPM group, the production of IL-12 could be enhanced to some extent, but compared with free CpG alone, the production capacity was lower, which shows the negligible effect to induce the IL-12 release (Fig. 4c). However, the secreted IL-12 levels stimulated by CSPM@CpG were significantly higher than that of CSP, CSPM, or CpG alone. The enhanced intracellular uptake and low nuclease degradation of CpG in this delivery system could be the direct causes. Because it was difficult for CpG alone to cross the cell membrane due to the negative charge and it was easily degraded by nucleases as mentioned above. Meanwhile, the cell apoptosis assay of DC cells incubated with CSPM@CpG under 650 nm and 808 nm laser irradiation revealed that irradiation had less effect on DC cells (Supplementary Fig. 11). The results demonstrate that CSPM@CpG exhibited great potential for CpG delivery and immunotherapy applications.

### In vivo biodistribution, photothermal imaging, and combined PTT-PDT-immune treatment with CSPM@CpG

**Fig. 5:**
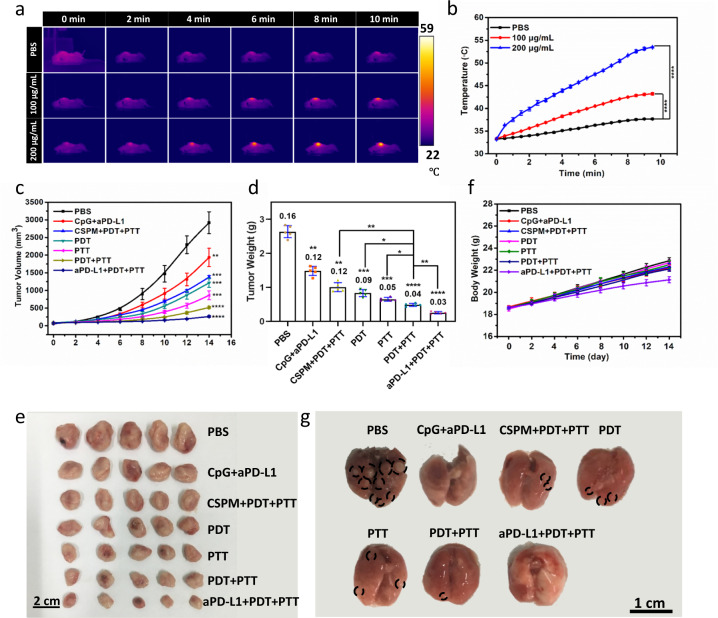
In vivo photothermal imaging and combined PTT-PDT-immunotherapy with CSPM@CpG. **a** In vivo infrared thermal photographs of PBS and CSPM@CpG (100 and 200 μg mL^−1^) recorded at different points when exposed to 808 nm laser irradiation. **b** Change of the temperature (tumor site) in vivo over a period of 10 min under PBS and CSPM@CpG (100 and 200 μg mL^−1^) treatment. **c** Tumor volumes of different groups of 4T1 tumor-bearing mice. **d** Histogram of mean tumor weight of different groups with various treatments on the 14th day. **e** Images of the excised tumors at the last day of the experiment with different treatments. **f** Body weight curves of mice during 14 day treatments. **g** Photographs of the lungs from different groups. The tissues in circles represent metastatic tumors. Data are presented as means ± standard deviation (s.d.) (*n* = 5). *p* values *<0.05, **<0.01, ***<0.001, and ****<0.0001.

Prior to studying anti-tumor efficiency, quantitative biodistribution of 4T1-bearing mice at four different time points (2 h, 6 h, 12 h, and 24 h) after intravenously injected CSPM@CpG was analyzed by determining the Cu content in the major organs (heart, liver, spleen, lung, and kidneys) and tumors (Supplementary Fig. 12). The content of Cu was found to be 7.07% of the injected dose per gram tissue (% ID/g) in the tumor at 2 h, and reached the maximum level at 12 h post-injection (24.29% ID/g), then began to decline, reaching (17.45% ID/g) at 24 h post-injection. Importantly, few uptake of CSPM@CpG by macrophages compared with 4T1 cells suggested that the accumulation in liver was not due to the uptake by the reticuloendothelial system, but the temporary retention caused by the liver’s unique anatomical and physiological structure (Supplementary Fig. 13). Next, the photothermal effect of CSPM@CpG in vivo was investigated. Briefly, tumor-bearing mice were exposed to 808 nm laser irradiation for 10 min at 12 h post-intravenous injection of CSPM@CpG and the temperature was recorded at selected time points by infrared thermal imaging camera (Fig. 5a). The local temperature of tumor sites in CSPM@CpG group at 200 μg mL^−1^ increased rapidly to higher than 58.6 °C, which was high enough to ablate tumor cells in PTT (Fig. 5b). By contrast, the tumor temperature of the PBS group showed a feeble increase under the same experimental condition. All these data demonstrated the excellent photothermal conversion of CSPM@CpG nanocomposites in vivo.

On the basis of the above-mentioned results, the tumor-inhibiting efficiency of CSPM@CpG in the tumor-bearing mice was further tested. We could observe that the tumor treated with PBS showed a rapid growth, while tumor growth was remarkably inhibited in the CSPM@CpG + PDT + PTT (abbreviated as PDT + PTT) group (Fig. 5c). For the mice treated with PDT or PTT, tumor growth exhibited some inhibition. The group treated with CpG and aPD-L1 had some inhibition for tumor growth after 14 days treatment, indicating that immunotherapy alone was weak for large solid tumors. By contrast, the tumor of CSPM@CpG + aPD-L1 + PDT + PTT (abbreviated as aPD-L1 + PDT + PTT) group was smaller than that of PDT + PTT group and CpG + aPD-L1 group, suggesting the synergistic therapeutic efficiency of CSPM@CpG nanocomposite. The tumor weight and the real images of tumor after different treatments on day 14, also demonstrated a remarkable inhibition of tumors treated with aPD-L1 + PDT + PTT group compared with PBS group (Fig. 5d, e). Simultaneously, mice body weights during the treatment period were also recorded to evaluate the potential long-term toxicity in vivo. The mice weights of all groups remained normal without any obvious decrease (Fig. 5f). In addition, the toxicity was further assessed by histological tissue imaging in major tissues (heart, liver, spleen, lung, and kidney) after various treatment through hematoxylin and eosin (H&E) staining. There were no appreciable histopathological abnormalities on mice from the combination therapy group, indicating that CSPM@CpG nanocomposite induced no apparent toxicity to mice (Supplementary Fig. 14). We further photographed the lung of the mice (Fig. 5g). The lungs became atrophic obviously and there were pulmonary metastasis of cancer cells occurred heavily in the PBS-treated mice. Additionally, no pulmonary metastasis was observed from the CpG + aPD-L1 group and aPD-L1 + PDT + PTT compared with other groups because of the activation of immune system by immunotherapy.

### Immunological responses after the combined PTT-PDT-Immune treatment

**Fig. 6:**
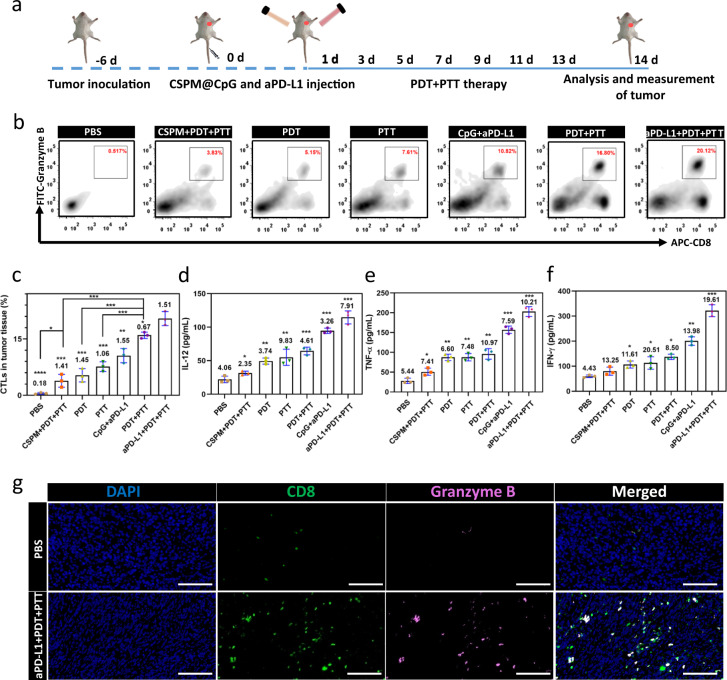
Immunological responses after combined PTT-PDT-immunotherapy with CSPM@CpG. **a** Schematic illustration of CSPM@CpG and aPD-L1 combination therapy. **b**, **c** Representative flow cytometry data (**b**) and proportion of CTLs infiltration in tumors (**c**) (CD8^+^ Granzyme B^+^ cells were defined as CTLs). **d**, **e** The production of IL-12 (**d**), TNF-α (**e**), and IFN-γ (**f**) in serum of mice with different treatments. **g** Representative immunofluorescence images of tumor tissues from PBS and aPD-L1 + PDT + PTT group. 2-(4-amidinophenyl)-6-indolecarbamidine dihydrochloride (DAPI)-labeled nuclei (blue); anti-CD8 + antibody-labeled T cells (green); anti-Granzyme B antibody-labeled T cells (pink). Scale bar: 100 μm. Data are presented as means ± standard deviation (s.d.; *n* = 5). *p* values *<0.05, **<0.01, ***<0.001, and ****<0.0001.

The effective inhibition of tumors in aPD-L1 + PDT + PTT group was not only the effect of PDT and PTT, but also the immunotherapy (Fig. 6a). To explore the infiltration of CTLs in tumor site, tumors harvested on day 14 with various treatments were analyzed by the flow cytometry and immunofluorescence. The results revealed that for PBS group, tumors had limited T cells infiltration. Comparatively, tumors treated with aPD-L1 + PDT + PTT were remarkably infiltrated by CTLs (CD8 + Granzyme B + cells were defined as CTLs) (Fig. 6b). The percentage of CTLs was ~40-fold of that in the PBS group and 2-fold or 1.2-fold compared with that in the group treated with CpG + aPD-L1 and PDT + PTT (Fig. 6c), respectively, owing to the capacity of aPD-L1 to block the PD-1/PD-L1 pathway and further prevent CTLs from dysfunction and exhaustion. To further investigate the activation of CTLs, secretion of cytokines in serum were detected through ELISA (Fig. 6d–f). For aPD-L1 + PDT + PTT group, tumor presented the highest level of IL-12, TNF-α, and IFN-γ, owing to the capability of IL-12 to induce the secretion of IFN-γ. The same outcomes of infiltration of CTLs in tumor site were observed through immunofluorescence (Fig. 6g and Supplementary Fig. 15). Tumors from aPD-L1 + PDT + PTT treated mice were considerably infiltrated by both CD8 + and Granzm B + T cells in comparison with limited infiltration of PBS group.

## Discussion

**Fig. 7:**
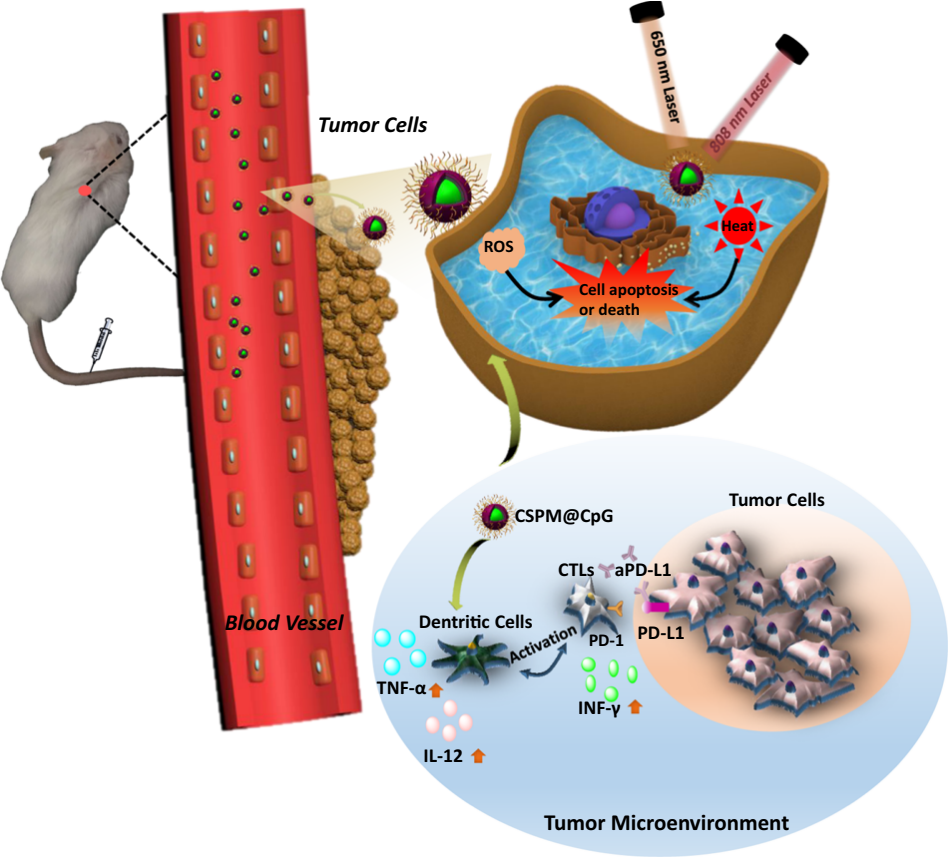
The proposed mechanism of anti-tumor and immune responses induced by CSPM@CpG in combination with aPD-L1 therapy. Briefly, CSPM@CpG got through the vessel and arrived at tumor microenvironment. Then CpG are specifically identified by Toll-like receptor 9 (TLR9) in plasmacytoid dendritic cells and thus stimulate an immune response to produce helper T cell 1 (Th1) and a pro-inflammatory cytokine TNF-α and IL-12. IL-12 could promote maturity of CTLs and infiltration in tumor site. At the same time, activated CTLs could secrete IFN-γ to kill tumor cells. However, tumor cells could induce CTLs exhaustion through PD-L1 signaling pathway for immune evasion, which could be effectively blockaded by aPD-L1 in turn. So PD-L1 antibody could bind with PD-L1 and remove immunosuppression to enhance immunotherapy. At the same time, the nanocomposite with efficient anti-tumor efficiency could effectively release lots of heat and ROS under 650 nm laser and 808 nm laser irradiation, causing apoptosis and necrosis of tumor cells.

As depicted in Fig. 7, PTT, PDT, and immunotherapy were well combined in our developed CSPM@CpG nanocomposite for their complementary advantages to achieve a synergistic treatment effect. The nanocomposite with efficient anti-tumor efficiency could effectively release lots of heat and ROS under laser irradiation, causing apoptosis and necrosis of tumor cells. Meanwhile, CSPM@CpG nanocomposite could ameliorate the hypoxic condition in the tumor site via inducing the decomposition of H_2_O_2_ to further promote photodynamic efficiency. More significantly, treatment with CSPM@CpG combined with aPD-L1 led to the activation of dendritic cells followed by secret of cellular cytokines and infiltration of CTLs. Besides eliminating primary tumor cells with the synergic effect of PTT, PDT, and immunotherapy, the enhanced immunotherapy with CSPM@CpG could inhibit the metastasis of tumor through the long-term immunological response of CpG.

In summary, we have successfully developed a multifunctional nanocomposite to load CpG based on Cu_9_S_5_ nanoparticles (CSPM@CpG) for curing solid tumors and inhibiting metastasis. The obtained nanocomposite exhibited excellent NIR activated therapeutic performance both in vitro and in vivo. Effective tumor growth suppression has been achieved from combination of CSPM@CpG-based PTT-PDT with aPD-L1 group under the light irradiation. Subsequent CTLs infiltration experiments demonstrate that the nanocomposite could also increase CpG uptake and enhance more production of immune cytokines, which effectively inhibited the metastasis of tumors. With excellent biocompatibility, our Cu_9_S_5_-based theranostic nanocomposite could be a promising candidate for combination phototherapy with immunotherapy in clinical applications.

## Methods

### Materials

Sodium diethyldithiocarbamate (SDEDTC), cetyltrimethylammonium bromide (CTAB), tetraethylorthosilicate (TEOS), and (3-aminopropyl) triethoxysilane (APTES) are analytically pure and were purchased from Aladdin Chemistry Co. Ltd., Shanghai, China. Anhydrous cupric chloride (CuCl_2_), protoporphyrin IX (PpIX), and poly(allylamine hydrochloride) (PAH) were obtained from Meryer Chemical Technology Co. Ltd, Shanghai, China. All of the other regents were obtained from Sinopharm Chemical Reagent Co. (Shanghai, China). CpG (1826) oligodeoxynucleotides (5′-TCCATGACGTTCCTGACGTT-3′) were synthesized by Sangon Biotechnology Co., Ltd., Shanghai, China. All reagents were used without further purification. Ultrapure water was used throughout all experiments and to prepare all solutions. Cell counting kit-8 (CCK-8) was purchased from Zoman Biotechnology Co, Ltd (Beijing, China). High glucose Dulbecco’s modified Eagle’s medium (DMEM), 1% penicillin–streptomycin (PS), 0.25% trypsin–EDTA, phosphate and phosphate-buffered saline (PBS) were obtained from Hyclone. Fetal bovine serum (FBS) was purchased from Gibco Invitrogen.

### Synthesis of copper diethyldithiocarbamate precursor

First, copper precursor solution (Cu(DEDTC)_2_) was prepared by reacting CuCl_2_ with SDEDTC as follows: a solution of SDEDTC in distilled water was added dropwise into another solution CuCl_2_ and distilled water under magnetic stirring at room temperature, forming a dark brown turbid solution, and then kept for 4 h of continuously stirring. Lastly, the dark brown product was obtained by filtration and dried under vacuum before use.

### Synthesis of oleylamine-capped Cu_9_S_5_ nanocrystals

Cu_9_S_5_ nanocrystals were synthesized by a thermal decomposition method according to a previously reported procedure^42^. In total, 15 mL of oleylamine in a three-necked flask (100 mL) was degassed at 80 °C in order to remove any water and oxygen and subsequently was slowly heated to 300 °C and kept at a constant temperature for 20 min under argon atmosphere. Then, another 5 mL of oleylamine containing 1 mmol of Cu(DEDTC)_2_ was rapidly injected into the above hot oleylamine, forming dark green solution immediately. After maintaining the temperature for 10 min, the solution was cooled to room temperature naturally. The resultant mixture was centrifugally separated with a centrifugal force of 12,000 rpm for 10 min and then was washed with hexane and ethanol twice. Finally, Cu_9_S_5_ nanocrystals were dispersed in 10 mL of chloroform for later use.

### Synthesis of CTAB modification of Cu_9_S_5_ nanocrystals

Briefly, the above 10 mL of chloroform solution containing the as-prepared Cu_9_S_5_ nanocrystals was added dropwise into 80 mL, 25 mg mL^−1^ of CTAB aqueous solution under stirring at 40 °C, forming a brown oil-in-water microemulsion. Subsequently, the microemulsion was stirred vigorously at 40 °C for 48 h, and then chloroform was completely evaporated from the mixture under rotary evaporation. Finally, the as-formed solution was diluted to 100 mL with distilled water and kept in the oven at 36 °C for further uses.

### Synthesis of Cu_9_S_5_@mSiO_2_-PpIX (CSP)

To synthesize CSP, PpIX was first modified with APTES by adding 6 mg of 1-(3-Dimethylaminopropyl)-3-ethylcarbodiimide hydrochloride (EDC•HCl), 4 mg of N-hydroxysuccinimide (NHS), and 12 µL of APTES into 2.0 mg of PpIX predisolved in 500 µL of dimethylsulfoxide (DMSO). Subsequently, the mixture was stirred at room temperature for 2 h at 800 rpm, then silica shell was coated to form CS nanospheres. In a typical procedure, 3 mL of ethanol was added to 50 mL of the above Cu_9_S_5_/CTAB solution, followed by continuous stirring for 1 h at 60 °C. Then 100 μL of NaOH (30 mg mL^−1^), subsequently, 100 μL of TEOS was immediately dropped into the solution, the reaction system stirring for another 1 h, obtaining a solution containing Cu_9_S_5_@mSiO_2_ core–shell nanocomposites. For coating PpIX conjugated mesoporous silica onto Cu_9_S_5_, 200 μL of PpIX modified with APTES was added to as-formed solution upon another 3 h of stirring, leading to a solution of PpIX modified Cu_9_S_5_@mSiO_2_ core–shell nanocomposites. The loading efficiency (LE) and the encapsulating efficiency (EE) of PpIX were calculated according to the following formula:

$${\mathrm{Loading}}\,{\mathrm{efficiency}}\,(\% ) = \frac{{{\mathrm{Weight}}\,{\mathrm{of}}\,{\mathrm{loaded}}\,{\mathrm{PpIX}}}}{{{\mathrm{Weight}}\,{\mathrm{of}}\,{\mathrm{CSP}}\,{\mathrm{NPs}}}} \times 100\%$$

$${\mathrm{Encapsulating}}\,{\mathrm{efficiency}}\,(\% ) = \frac{{{\mathrm{Weight}}\,{\mathrm{of}}\,{\mathrm{loaded}}\,{\mathrm{PpIX}}}}{{{\mathrm{Weight}}\,{\mathrm{of}}\,{\mathrm{devoted}}\,{\mathrm{PpIX}}}} \times 100\%$$

After that, the mixture was centrifuged and washed with ethanol to remove the impurities. Finally, the obtained product was redispersed in 60 mL acetone and refluxed at 80 °C for 48 h to remove the template CTAB according to the method reported by Zhao et al.^41^.

### Synthesis of Cu_9_S_5_@mSiO_2_-PpIX@MnO_2_ (CSPM)

In total, 5 mL of KMnO_4_ solution (50 μg mL^−1^) was added into 2 mL of CSP suspension (1 mg mL^−1^) under ultrasonication drop by drop. After 5 min, CSPM was obtained by centrifugation at 10,000 rpm and washed with water for three times.

### Synthesis of Cu_9_S_5_@mSiO_2_-PpIX@MnO_2_@CpG (CSPM@CpG) nanocomposite

In all, 5 mL, 4 mg mL^−1^ of CSPM solution was added to 10 mL, 5 mg mL^−1^ of PAH solution under ultrasonication and stirred continuously for 2 h. The resultant solution was centrifuged and washed with water for three times. After that, the solution was added into CpG solution under magnetic stirring at ice-bath for 2 h. The solution was centrifuged, washed with water for three times and then freeze-dried in vacuum for further experiments.

### Characterizations

Powder X-ray diffraction (XRD) patterns were conducted by a D8 advance (Bruker, Germany). The sizes and morphologies of samples were determined by a transmission electron microscope (TEM) at an accelerating voltage of 200 KV (JEM-2100, JEOL, Japan). The specific surface area and pore volume of the products were determined by Brunauer–Emmett–Teller (BET) and Barett–Joyner–Halenda (BJH) methods (TriStar 3020, Micromeritics, America). X-ray-photoelectron spectroscopy (XPS) analysis was examined with kratos Axis ultra dld. UV-visible absorption spectra were recorded by a Hitachi U-2900 Spectrophotometer. Inductively coupled plasma optical emission spectroscopy (ICP-OES, PE 8300) was used to validate the content of copper ions intracellular uptake by cells. The Zetasizer Nano Z (Malvern, Britain) was selected to measure zeta potential.

### Drug release

Briefly, 5 mg lyophilized NPs were put in a centrifuge tube and redispersed in 10 ml phosphate buffer solution (PBS, pH 7.4 and 5.0). The tube was put into an orbital shaker water bath and vibrated at 120 rpm at 37 °C or 43 °C. At designated time intervals, the tube was taken out and centrifuged at 10,000 rpm for 5 min. Then, the supernatant was tested for CpG concentration using Nanodrop. The pellet was resuspended in 10 mL fresh PBS solution and put back into the shaker for subsequent measurement. The cumulative release of CpG from NPs was plotted against time.

### Photothermal and photodynamic performance under NIR laser irradiation

To investigate the photothermal effect of CSPM@CpG nanocomposites, various concentrations (0, 50, 100, 200, and 500 µg mL^−1^) were added to a cuvette and irradiated with 808 nm NIR laser at a power density of 2 W cm^−2^ for 5 min. The temperature of the solution was measured in 30 seconds intervals with LTX3-P infrared Imaging (DALI TECHNOLOGY, Zhejiang, China).

The singlet oxygen generation ability by CSPM@CpG NPs under the 650 nm irradiation was detected using the chemical probe -2′,7′-Dichlorofluorescein (DCFH). Briefly, 20 μL DCFH solution (2 mM) was added to 2 mL CSPM@CpG solutions (100 µg mL^−1^). With the irradiation of the 650-nm NIR laser (1 W cm^−2^) for 5 min, the increase in the fluorescence intensity of DCF (*λ*_Em_ = 488 nm) was recorded every minute with a fluorescence spectrophotometer (Hitachi F-7000). For comparison, the solution of DCFH (2 mM) was used for control experiments under the same condition.

### Catalytic activity of CSPM@CpG

The catalytic-like activity of CSPM@CpG was evaluated by observing the decomposition of hydrogen peroxide. The tubes containing (1) H_2_O_2_ (10 mM), (2) CSPM@CpG (100 μg mL^−1^) + H_2_O_2_ (10 mM) respectively, were reacted at room temperature for 30 min, and UV-Vis spectra were recorded at intervals of 10 min. Besides, to investigate the influence of pH on the H_2_O_2_ decomposition, the experiment was carried out by adding CSPM@CpG (100 μg mL^−1^) in PBS (pH 6.5) containing H_2_O_2_ (10 mM) at room temperature. H_2_O_2_ (10 mM) in PBS (pH 6.5) was performed as comparison.

### In vitro cell experiments

4T1 murine breast cancer cell were obtained from ATCC (ATCC^®^ CRL-2539^TM^) and not listed in the database of commonly misidentified cell lines, ICLAC (the cell lines were not tested for mycoplasma contamination). Then 4T1 cells were cultured in a high glucose DMEM medium supplemented with 10% FBS and 1% PS at 37 °C in a fully humidified atmosphere with 5% CO_2_. The cells were seeded in 96-well at 8 × 10^3^ cells per well with 100 μL high glucose DMEM medium. After incubated for 24 h, 4T1 cells were treated by different concentrations of CSPM@CpG (ranging from 2.5 to 1000 μg mL^−1^) for another 24 h. In all, 100 μL of DMEM (without FBS) containing 10% CCK8 was added into each well. After incubation for 4 h at 37 °C, the absorbance at 450 nm was measured using a standard enzyme linked immunosorbent assay (ELISA) format spectrophotometer. Experiment was repeated three times and the cell viability percentage was calculated based on the average of each experiment.

To quantify the cellular uptake efficiencies of CSPM@CpG, 4T1 cells were seeded in a 24-well plate at a density of 2 × 10^4^ per well and incubated for 24 h, then 800 µL different concentrations of CSPM@CpG (0, 25, 50, and 100 µg mL^−1^) was added into well. After incubation for different time periods, the cells were collected and washed three times with PBS. Then 4T1 cells were resuspended into 500 μL PBS and digested with aqua regia to quantify the content of Cu with ICP. Additionally, 4T1 cells were incubated with CSPM@CPG (100 µg mL^−1^) for different time periods, then harvested by trypsinization, and were visualized under a confocal laser scanning microscope (CLSM, Leica TCS SP5 II).

The intracellular reactive oxygen species generation of CSPM@CpG NPs with 650 nm laser irradiation was also detected using DCFH-DA Reactive Oxygen Species Assay Kit. 4T1 cells were plated at 5 × 10^4^ cells per well on confocal culture dish until cell attachment. Following incubation with different centration of CSPM@CpG NPs (25, 50, 100, and 200 µg mL^−1^) for 4 h at 37 °C, the cells were washed with PBS for three times and then proper amount of DCFH-DA was added into the wells. After incubation for another 0.5 h, the cells were irradiated under 650 nm laser with the power intensity of 1 W cm^−2^ for 5 min. Finally, the cell imaging was captured by using CLSM with an excitation of 488 nm and emission of 530 nm. The generation of ROS in 4T1 cells without adding nanocomposite under 655 nm laser irradiation for 5 min was taken as control.

The production of IL-12 by the dendritic cells 2.4 (DCs 2.4) under different treatments (CpG, CSP, CSPM, and CSPM@CpG) was quantified by mouse IL-12 precoated ELISA kit and mouse IL-12 precoated ELISA kit (Lianke Bio). DC 2.4 cells were seeded in transwell plates at a density of 1 × 10^6^ cells per well overnight and then incubated with free CpG, CSP, CSPM, and CSPM@CpG for 12 h, respectively. Then lower supernatant solution was collected and detected by ELISA, using antibody pair specific to these cytokines following the protocol recommended by the manufacturer.

To evaluate the synergic therapeutic efficacy of CSPM@CpG NPs as a PDT/PTT/immunotherapy agent, the cell cytotoxicity was measured with CCK-8 assay. Briefly, 4T1 cells seeded in 96-well plates were incubated with different concentration of NPs (0, 25, 50, 100, and 200 μg mL^−1^) for 12 h. For PDT, PTT, and PDT + PTT groups, 4T1 cells were treated with CSPM NPs. After they were washed three times by PBS to remove the excess material, 4T1 cells were exposed to 650 nm (1 W cm^−2^) for 5 min for PDT and irradiated with 808 nm (2 W cm^−2^) laser for PTT, respectively. PDT + PTT group was conducted under 808 nm and 650 nm irradiation for 5 min simultaneously. Immunotherapy group was performed through adding CpG into wells. PDT + PTT + CpG group was treated with CSPM@CpG and irradiated under 650 nm and 808 nm irradiation for 5 min at the same time. Then 100 μL of DMEM (without FBS) containing 10% CCK8 was added into each well. To quantitatively measure the number of 4T1 cells survived after various treatments, the cell viability was also measured by CCK8 assay as described above. Cells without treatment were used as control groups for comparison. The synergistic effect of PTT-PDT-immunotherapy was evaluated by combination index (CI) analysis. CI > 1 denotes antagonism; CI = 1 additivity, and CI < 1 synergism^49^.

Cells apoptosis was detected by using an Annexin-V-FITC Staining kit. 4T1 cells were placed into 24-well plates at a density of 2 × 10^4^ cells per well and allowed to adhere prior to addition of CSPM@CpG. After 4 h incubation, cells were irradiated under different treatment: no treatment; 650 nm laser irradiation at a power density of 1 W cm^−2^ for 5 min; 808 nm irradiation at 2 W cm^−2^ for 5 min; 650 nm and 808 nm simultaneous irradiation at 1 W cm^−2^ and 2 W cm^−2^, respectively for 5 min and then incubated for a further 12 h. Cells were digested, collected, and stained with 5 μL recombinant human anti-Annexin-V-FITC and 5 μL of propidium iodide (PI). After reaction at room temperature in the dark for 10 min, all the samples were immediately detected by using flow cytometry (BD FACSVerse).

### Animal models

Female Balb/c mice (4–5 weeks) were purchased from Shanghai Laboratory Animal Center (SLAC, Shanghai, China) and bred in a sterilized, specific pathogen-free (SPF) Lab of Tongji University. Then the 5 × 10^5^ 4T1 cells were injected into the left mammary fat pad of 5-week-old female BALB/c mice. All animal procedures were conformed to the Guide for the Care and Use of Laboratory Animals and animal study protocols were approved by Tongji University Experimental Animal Center.

### In vivo photothermal imaging and biodistribution

For in vivo photothermal effect evaluation, 4T1 tumor-bearing Balb/c mice were injected with 100 μL of CSPM@CpG suspension (100 and 200 µg mL^−1^, respectively) and irradiated with 808 nm NIR laser at the power density of 2 W cm^−2^ for 5 min after 12 h post-intravenous injection. The infrared thermal images of mice were obtained by the LTX3-P infrared Imaging for a designated time period. The tumor-bearing mice injected with PBS were selected as the control.

In order to study the biodistribution of CSPM@CpG, 4T1 tumor-bearing mice were injected intravenously with CSPM@CpG (450 μg mL^−1^, 0.15 mL) and killed after 2 h, 6 h, 12 h, and 24 h. The heart, liver, spleen, lung, kidney, and tumor were weighed and digested in aqua regia. Finally, the Cu content per gram of each organ was measured by ICP.

### In vivo animal experiments

After the tumors grew to a size of 80 mm^3^, the tumor-bearing mice were randomly assigned to six groups (*N* = 4 per group) and treated differently: (1) PBS; (2) CpG and PD-L1 antibody (1000 μg/Kg) (*InVivo*MAb anti-mouse PD-L1 (B7-H1), catalog number: BE0101; concentration: 5.8 mg/mL) injected percutaneously in advance; (3) CSPM exposed to 650 nm and 808 nm laser; (4) CSPM@CpG exposed to 650 nm laser with the power intensity of 1 W cm^−2^ for 5 min (PDT); (5) CSPM@CpG irradiated by 808 nm laser at a power intensity of 2 W cm^−2^ for 5 min (PTT); (6) CSPM@CpG irradiated by 650 nm laser (1 W cm^−2^, 5 min) and 808 nm laser (2 W cm^−2^, 5 min) simultaneously (PDT + PTT); (7) CSPM@CpG with 650 nm laser and 808 nm laser irradiation (the power intensity and irradiation time is the same as that mentioned above) and PD-L1 antibody (1000 μg/Kg) injected percutaneously in advance (aPD-L1 + PDT + PTT); Briefly, CSPM@CpG dissolved in 0.15 mL of PBS buffer solution at a concentration of 450 μg mL^−1^, were injected via the tail vein every other day for 2 weeks. Mice weight and tumor sizes were measured every 2 days. Tumor volume was calculated as tumor length × (tumor width)^2^ × 1/2 and tissue samples were collected for H&E staining.

Tumors were collected and fixed with 4% paraformaldehyde after 14 days treatment. Subsequently, tumor sections were dehydrated with different concentration of ethanol, blocked with BSA for 30 min, and incubated with individual primary antibodies against CD8 (FITC-conjugated anti-mouse CD8^+^; catalog number: 11-0081-82; concentration: 0.5 mg/mL) or Granzyme B (PE-conjugated anti-mouse Granzyme B; catalog number: ab225471; concentration: 0.5 mg/mL) overnight at 4 °C, followed by incubation with dye-conjugated secondary antibodies for 1 h at room temperature. After staining with DAPI for another 10 min, the sections were then washed thrice with PBS and observed under CLSM.

Tumors of different groups were harvested, gently grounded with the rubber end of a syringe and filtered through nylon mesh filters to acquire single-cell suspensions. The single-cell suspension was incubated with anti-CD16/32 (Anti-mouse CD16/32; catalog number: 08212-20-100; concentration: 0.5 mg/mL) to block nonspecific antibody binding. Cells were further stained by immunophenotyping antibodies with anti-CD8 antibody and anti-Granzyme B antibody. Subsequently, cells were stained with Fixable Viability Dye eFluor 450 and observed using BD flow cytometry. Data were analyzed using FlowJo 7.6.1.

### Statistics and reproducibility

All data are based on at least three independent experiments. The data were presented as the mean ± standard deviation. One-way single factorial analysis of variance (ANOVA) was performed to determine the statistical significance of the data. The statistical significance of the differences was expressed as *p* values *<0.05, **<0.01, ***<0.001, and ****<0.0001. All experiments have been reproduced and results confirmed.

### Reporting summary

Further information on research design is available in the Nature Research Reporting Summary linked to this article.

## Supplementary information

Supplementary Information

Description of Additional Supplementary Files

Supplementary Data 1

Reporting Summary

## Data Availability

The data used to generate the main results shown in Figs. 1–6 are available in Supplementary Data 1. All other data underlying the findings in the manuscript are available from the corresponding authors on reasonable request.
