# Supplementary material for: A Cu9S5 nanoparticle-based CpG delivery system for synergistic photothermal-, photodynamic- and immunotherapy
Source: Commun Biol. 2020 Jul 3;3:343. doi: 10.1038/s42003-020-1070-6 (PMC7334227; doi:10.1038/s42003-020-1070-6)
Supplement: Supplementary file 1 — Supplementary Information [file 42003_2020_1070_MOESM1_ESM.pdf]

Supporting Information

A Cu_9S_5 nanoparticle-based CpG delivery system for synergistic photothermal-, photodynamic- and immunotherapy

Lulu Zhou[1,2], Lv Chen[1,2], Xiaochun Hu[1], Yonglin Lu[1], Wenjie Liu[1], Yanting Sun[1],Tianming Yao[1], Chunyan Dong[1] & Shuo Shi[1]

[1] Shanghai Key Laboratory of Chemical Assessment and Sustainability, School of Chemical Science and Engineering, Breast Cancer Center, Shanghai East Hospital, Tongji University, Shanghai, 200092, P. R. China. [2]Theses authors contributed equally: Lulu Zhou, Lv Chen. Correspondence and requests for materials should be addressed to S.S. (email:shishuo@tongji.edu.cn) or C.D. (email: cy_dong@tongji.edu.cn).

Supplementary Figures

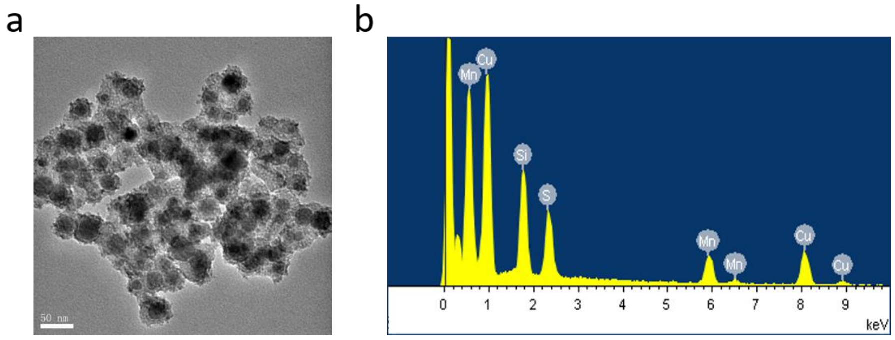

Supplementary Fig. 1 The morphology (**a**) and EDS spectrum (**b**) of CSPM@CpG nanocomposite.

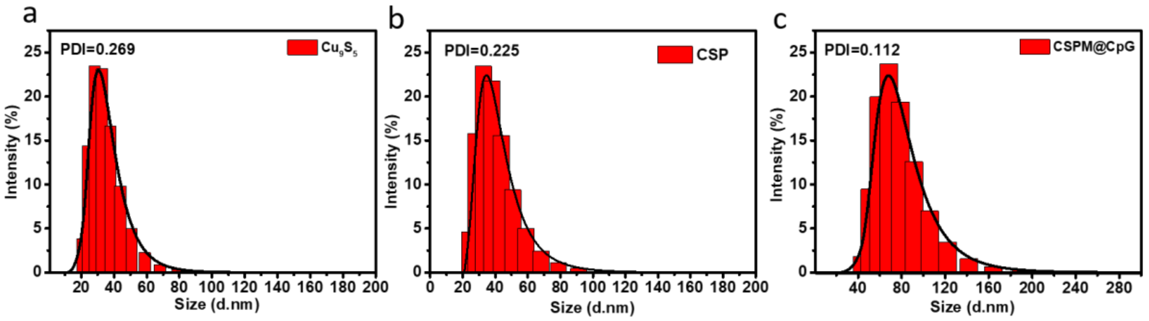

Supplementary Fig. 2 The size distributions of Cu_9S_5 (**a**), CSP (**b**) and CSPM@CpG (**c**).

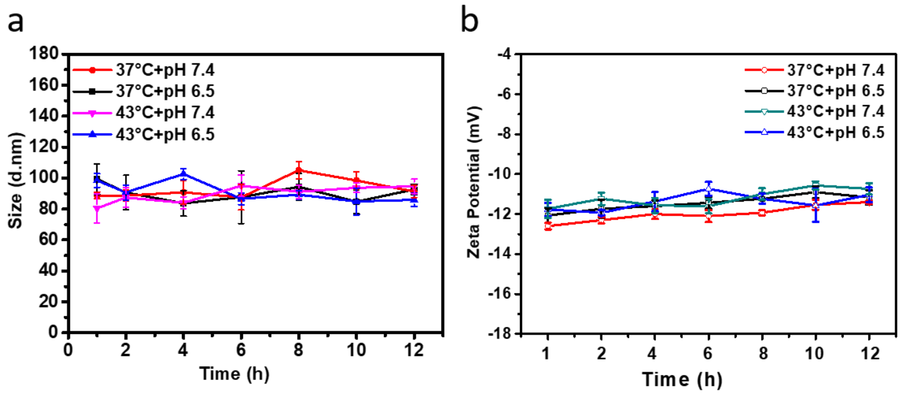

Supplementary Fig. 3 The sizes (**a**) and zeta-potential (**b**) of CSPM@CpG at different pH values at 37 ℃ or 43 ℃ within 12 h.

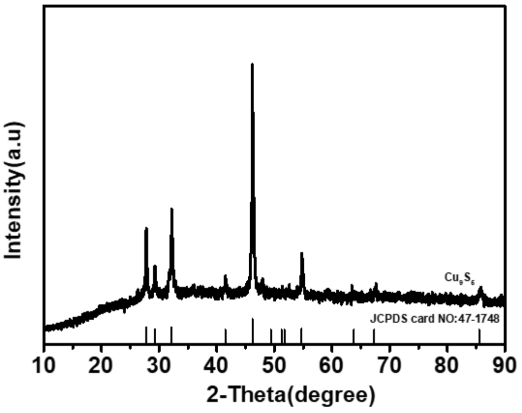

Supplementary Fig. 4 XRD pattern of oleylamine-capped Cu_9S_5 and standard Cu_9S_5.

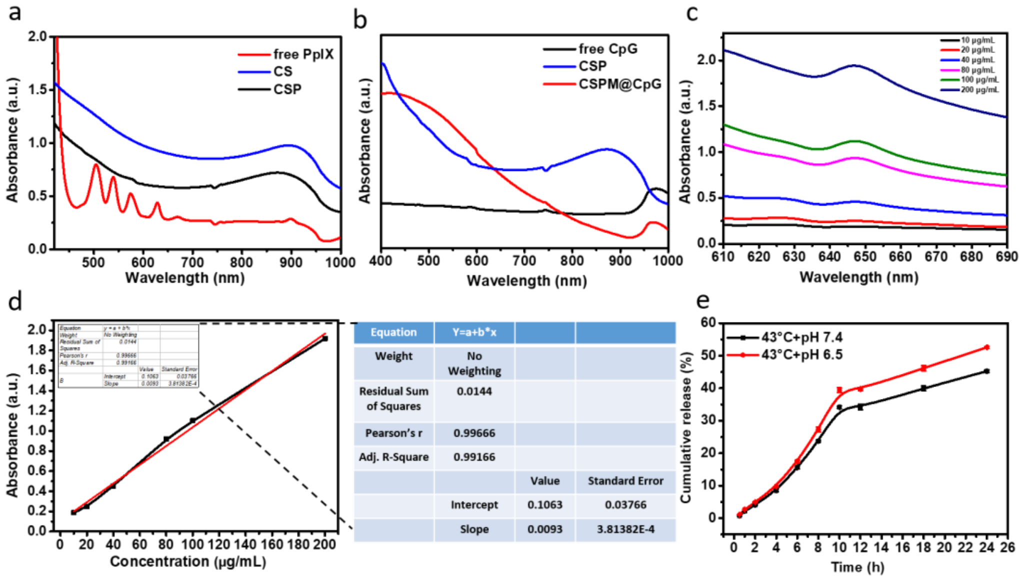

Supplementary Fig. 5 Uv-vis adsorption spectra of PpIX, CS and CSP (**a**) and CpG, CSPM@CpG (**b**). Uv-vis adsorption spectra of PpIX at different concentrations (**c**) and the standard absorption curve of PpIX at 650 nm (**d**). Percentage of cumulative released CpG from CSPM@CpG at different pH values (7.4 and 6.5) at 43 ℃ (**e**).

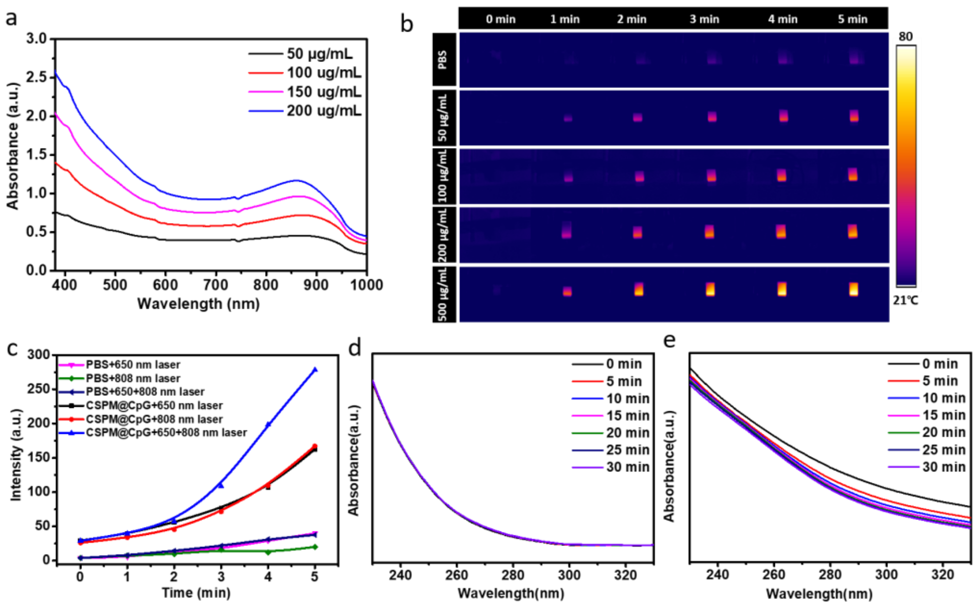

Supplementary Fig. 6 UV-vis adsorption spectra of CSP at different concentrations (**a**). In vitro infrared thermal photographs of CSPM@CpG recorded at different time intervals when exposed to 808 nm laser irradiation (**b**). ROS detection of CSPM@CpG nanocomposites (100 μg•mL^{-1}) with DCFH under 650 nm and 808 nm laser irradiation for 5 min (**c**). UV-Vis spectra of remainder H_2O_2 were recorded after reaction with PBS (**d**) and CSPM@CpG (**e**) for different times in pH 6.5, respectively.

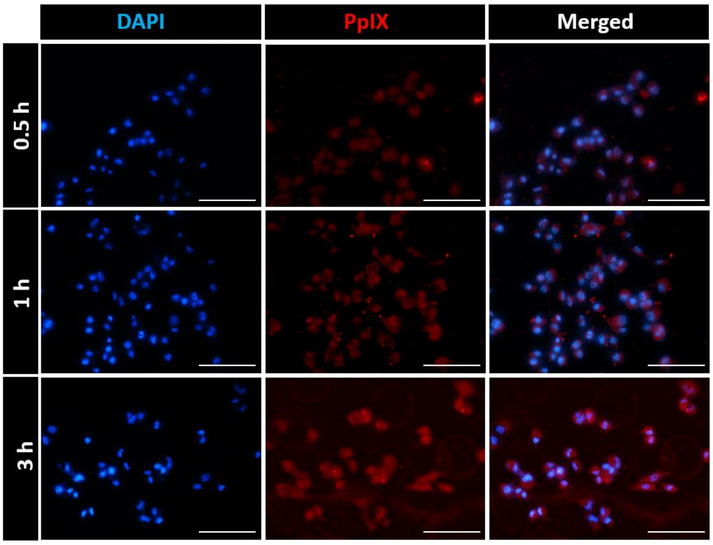

Supplementary Fig. 7 CLSM images of 4T1 cells incubated with CSPM@CpG for 0.5 h, 1 h and 3 h. DAPI for nuclei staining (blue) and PpIX fluorescence (red) were recorded. Scale bar: 100 μm.

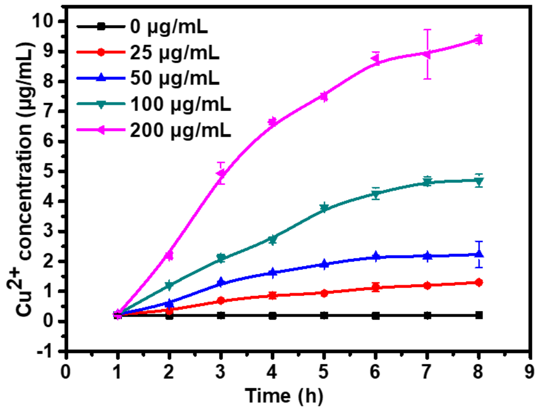

Supplementary Fig. 8 The cellular uptake by evaluating the content of Cu internalized by 4T1 cells at incubation with CSPM@CpG for different time periods through ICP detection. Date are presented as means ± standard deviation (s.d.) (n = 3).

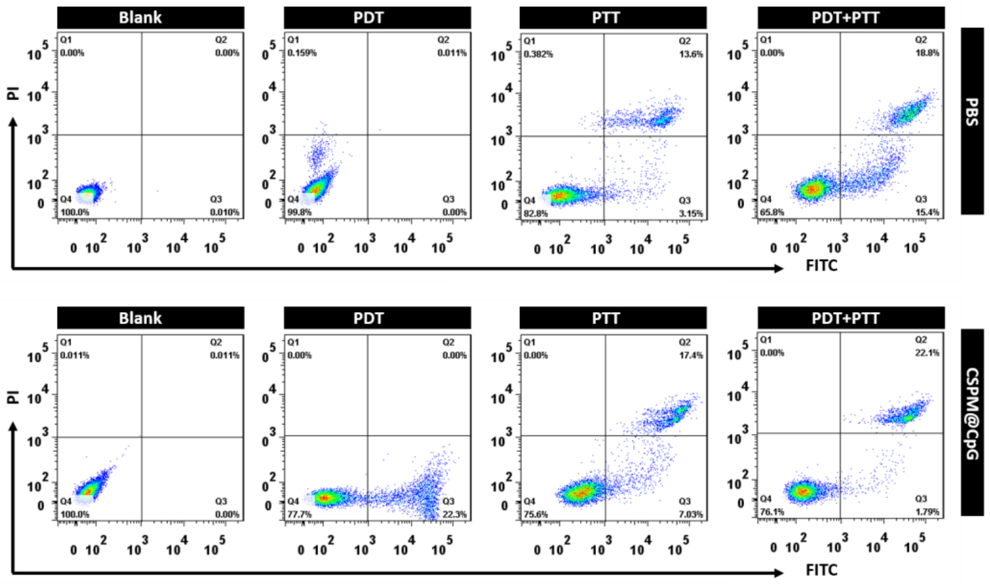

Supplementary Fig. 9 Flow cytometry analysis of 4T1 cells treated with PBS and CSPM@CpG (100 ug•mL^{-1}) under different NIR irradiation. In the case of early apoptosis (bottom right), Annexin V-Alexa Fluor 488 is positive and PI is negative. For late apoptosis (top right), both are positive. The dead cells (top left) have negative Annexin V-Alexa Fluor 488 and positive PI whereas in the case of live cells (bottom left) both are negative.

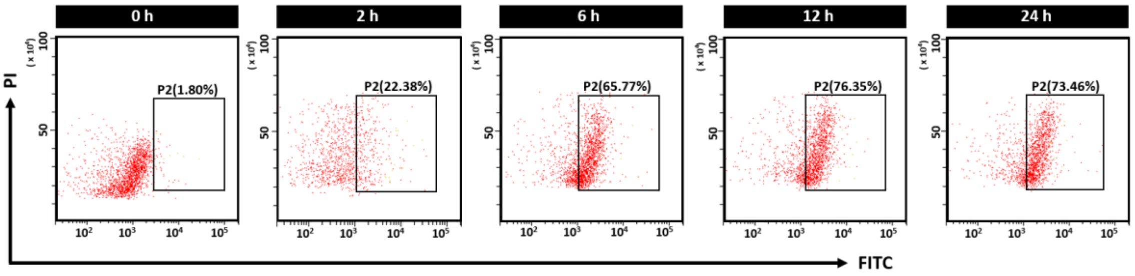

Supplementary Fig. 10 Flow cytometric assay of pDCs incubated with CSPM@CpG (100 μg/mL) for 0, 2, 6, 12 and 24 h in vitro.

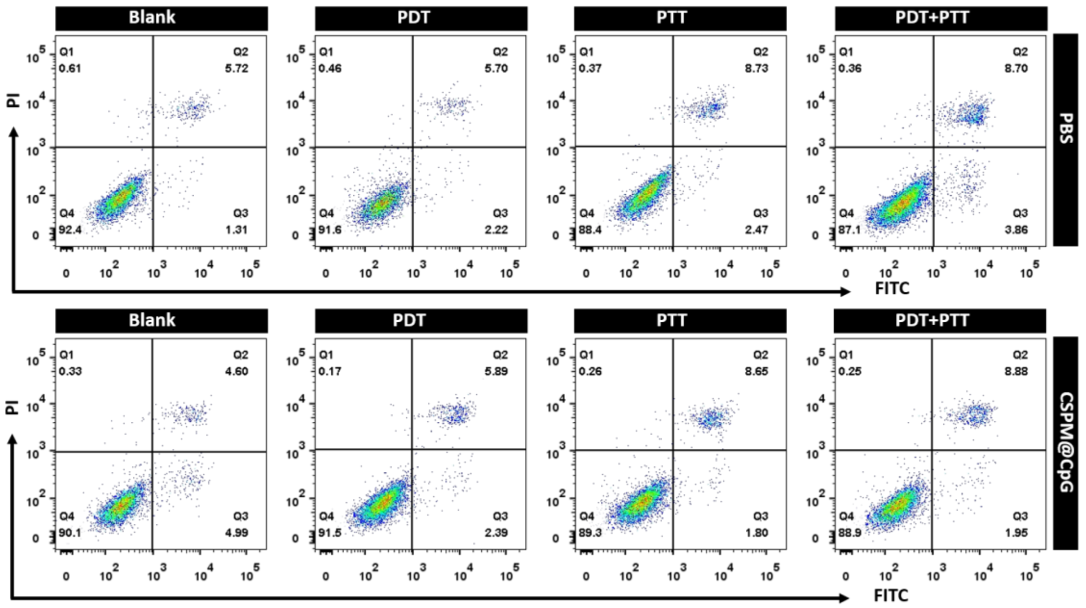

Supplementary Fig. 11 Flow cytometry analysis of DC cells treated with PBS and CSPM@CpG (100 ug•mL^{-1}) under different NIR irradiation. In the case of early apoptosis (bottom right), Annexin V-Alexa Fluor 488 is positive and PI is negative. For late apoptosis (top right), both are positive. The dead cells (top left) have negative Annexin V-Alexa Fluor 488 and positive PI whereas in the case of live cells (bottom left) both are negative.

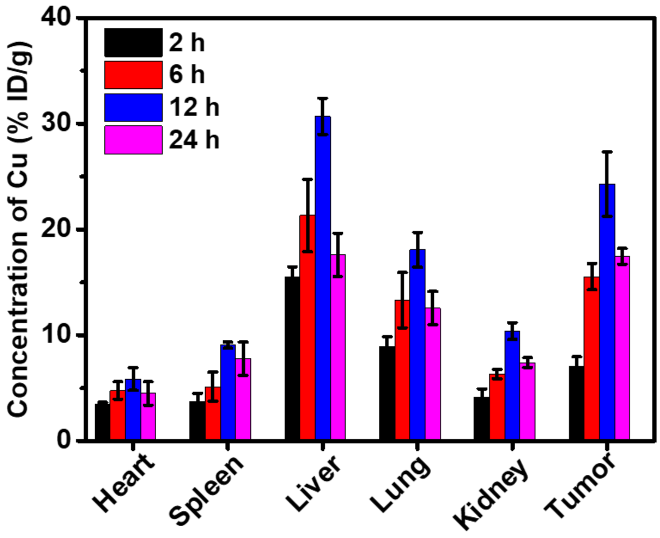

Supplementary Fig. 12 Biodistribution of Cu in major organs of tumor-bearing mice after injection of CSPM@CpG intravenously at different time points. Date are presented as means ± standard deviation (s.d.) (n = 3).

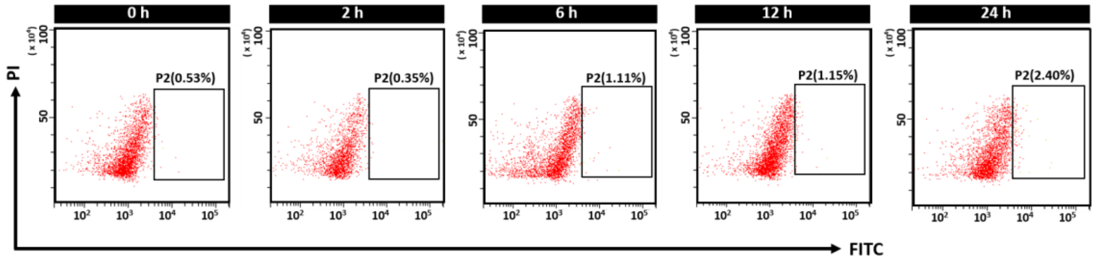

Supplementary Fig. 13 Flow cytometric assay of macrophages incubated with CSPM@CpG (100 μg/mL) for 0, 2, 6, 12 and 24 h in vitro.

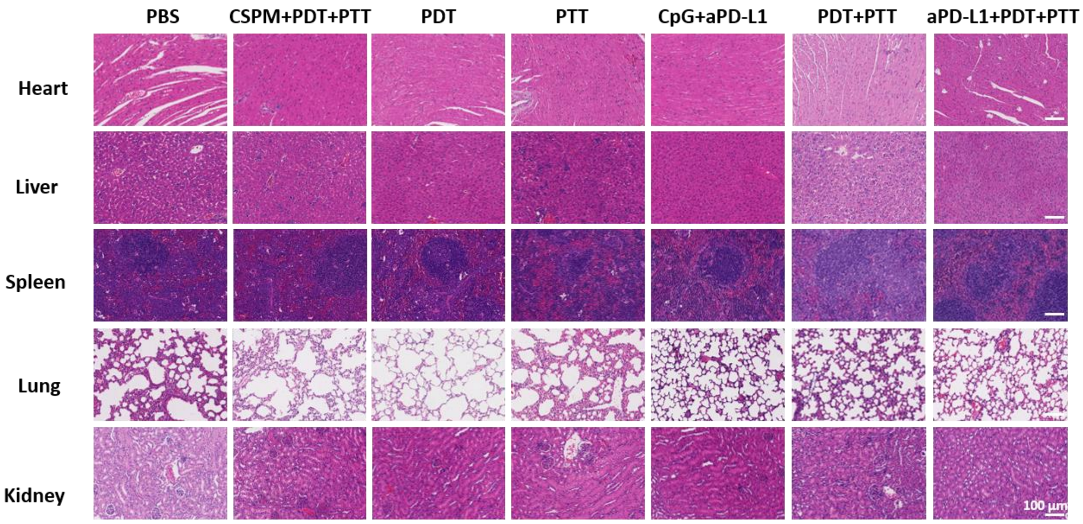

Supplementary Fig. 14 H&E staining of the major tissues after 14d treatment for PBS, CpG + aPD-L1, CSPM + PDT+ PTT, PDT, PTT, PDT + PTT and aPD-L1 + PDT + PTT. Scale bar:100 μm.

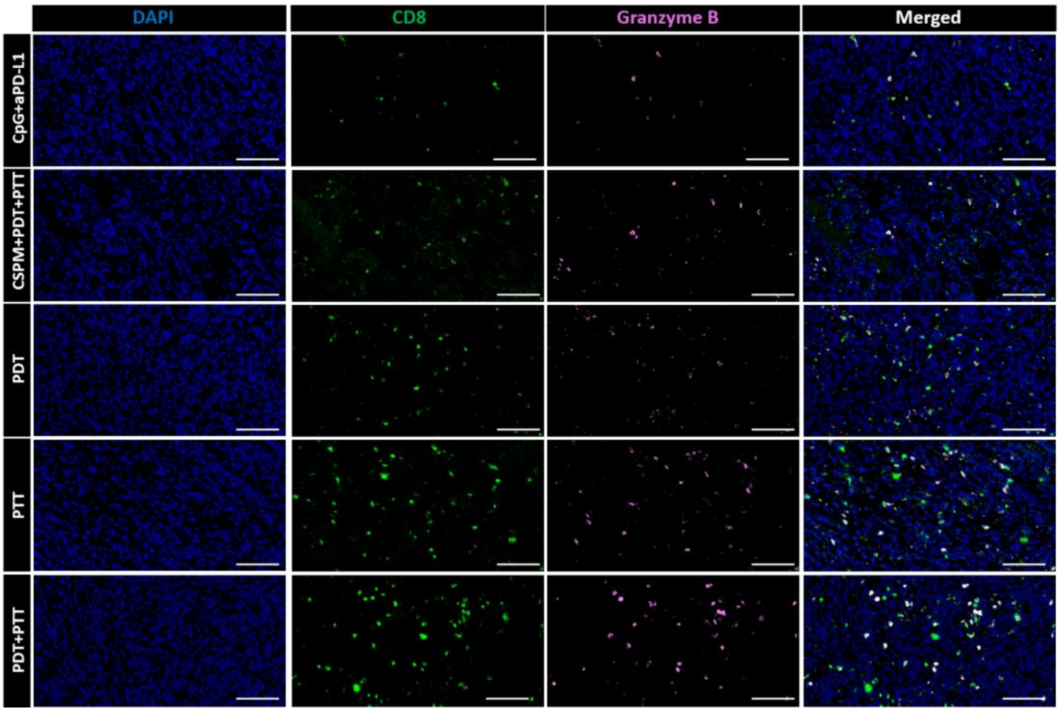

Supplementary Fig. 15 Representative immunofluorescence images of tumor tissues from different groups. 2-(4-amidinophenyl)-6-indolecarbamidine dihydrochloride (DAPI)-labeled nuclei (blue); anti-CD8+ antibody-labeled T cells (green); anti-Granzyme B antibody-labeled T cells (pink). Scale bar:100 μm.

Supplementary Tables

Table S1. The change of CpG concentration and zeta-potential of CSPM@CpG before and after 650 nm, 808 nm and 650 + 808 nm laser irradiation for 5 min, respectively.

	650 nm laser		808 nm laser		650 + 808 nm laser	
	Before	After	Before	After	Before	After
Concentration(μg/mL)	1.76	1.61	1.76	1.76	1.76	1.66
Zeta-Potential (mV)	-12.9	-11.1	-12.9	-13.0	-12.9	-12.8
