# Supplementary material for: A Cu9S5 nanoparticle-based CpG delivery system for synergistic photothermal-, photodynamic- and immunotherapy
Source: Commun Biol. 2020 Jul 3;3:343. doi: 10.1038/s42003-020-1070-6 (PMC7334227; doi:10.1038/s42003-020-1070-6)
Supplement: Supplementary file 2 — Description of Additional Supplementary Files [file 42003_2020_1070_MOESM2_ESM.pdf]

Description of additional supplementary files

Supplementary Data 1: The source data behind figures 1-6.
