# Supplementary material for: A Cu9S5 nanoparticle-based CpG delivery system for synergistic photothermal-, photodynamic- and immunotherapy
Source: Commun Biol. 2020 Jul 3;3:343. doi: 10.1038/s42003-020-1070-6 (PMC7334227; doi:10.1038/s42003-020-1070-6)
Supplement: Supplementary file 4 — Reporting Summary [file 42003_2020_1070_MOESM4_ESM.pdf]

nature research

Corresponding author(s): Shuo Shi
Last updated by author(s): Jun 5, 2020

Reporting Summary

Nature Research wishes to improve the reproducibility of the work that we publish. This form provides structure for consistency and transparency in reporting. For further information on Nature Research policies, see our Editorial Policies and the Editorial Policy Checklist.

Statistics

For all statistical analyses, confirm that the following items are present in the figure legend, table legend, main text, or Methods section.

n/a	Confirmed	
☐	☒	The exact sample size (*n*) for each experimental group/condition, given as a discrete number and unit of measurement
☐	☒	A statement on whether measurements were taken from distinct samples or whether the same sample was measured repeatedly
☐	☒	The statistical test(s) used AND whether they are one- or two-sided<br>*Only common tests should be described solely by name; describe more complex techniques in the Methods section.*
☐	☒	A description of all covariates tested
☐	☒	A description of any assumptions or corrections, such as tests of normality and adjustment for multiple comparisons
☐	☒	A full description of the statistical parameters including central tendency (e.g. means) or other basic estimates (e.g. regression coefficient) AND variation (e.g. standard deviation) or associated estimates of uncertainty (e.g. confidence intervals)
☒	☐	For null hypothesis testing, the test statistic (e.g. *F*, *t*, *r*) with confidence intervals, effect sizes, degrees of freedom and *P* value noted<br>*Give P values as exact values whenever suitable.*
☒	☐	For Bayesian analysis, information on the choice of priors and Markov chain Monte Carlo settings
☒	☐	For hierarchical and complex designs, identification of the appropriate level for tests and full reporting of outcomes
☒	☐	Estimates of effect sizes (e.g. Cohen's *d*, Pearson's *r*), indicating how they were calculated

Our web collection on statistics for biologists contains articles on many of the points above.

Software and code

Policy information about availability of computer code

Data collection	Powder X-ray diffraction (XRD) patterns were conducted by a D8 advance (Bruker, Germany). The sizes and morphologies of samples were determined by a transmission electron microscope (TEM) at an accelerating voltage of 200 KV (JEM-2100, JEOL, Japan). The specific surface area and pore volume of the products were determined by Brunauer-Emmett-Teller (BET) and Barett-Joyner-Halenda (BJH) methods (TriStar 3020, Micromeritics, America). X-ray-photoelectron spectroscopy (XPS) analysis was examined with kratos Axis ultra dld. UV-visible absorption spectra were recorded by a Hitachi U-2900 Spectrophotometer. Inductively coupled plasma optical emission spectroscopy (ICP-OES, PE 8300) was used to validate the content of copper ions intracellular uptake by cells. The Zetasizer Nano Z (Malvern, Britain) was selected to measure zeta potential.2D imaging data were obtained through Confocal Laser Scanning Microscope (Nikon, Japan). Flow cytometry (BD FACS Aria II, USA) is used to collect the flow cytometry data.
Data analysis	Data analysis, visualization, and statistical analyses were carried out with origin (8.0) ,flowjo(10), SPSS(V21.0) and Graph Pad.

For manuscripts utilizing custom algorithms or software that are central to the research but not yet described in published literature, software must be made available to editors and reviewers. We strongly encourage code deposition in a community repository (e.g. GitHub). See the Nature Research guidelines for submitting code & software for further information.

Data

Policy information about availability of data

All manuscripts must include a data availability statement. This statement should provide the following information, where applicable:
- Accession codes, unique identifiers, or web links for publicly available datasets
- A list of figures that have associated raw data
- A description of any restrictions on data availability

All other remaining data are available within the article and supplementary files, or available from the authors upon request.

Field-specific reporting

Please select the one below that is the best fit for your research. If you are not sure, read the appropriate sections before making your selection.

☒ Life sciences ☐ Behavioural & social sciences ☐ Ecological, evolutionary & environmental sciences

For a reference copy of the document with all sections, see nature.com/documents/nr-reporting-summary-flat.pdf

Life sciences study design

All studies must disclose on these points even when the disclosure is negative.

Sample size	The tumor diameters were measured by vernier caliper for three times to get mean value.
Data exclusions	Abnormal Data obtained from enzyme linked immunosorbent assay were excluded .
Replication	Measurement of Cytokines (such as TNF- α, IL-12 and INF-γ) were taken by protocols.
Randomization	To evaluate the anti-tumor effects in our tumor model, BALB/c mice were injected s.c. with 4T1 mammary tumor cells or PBS and randomly assigned to either of the study groups (n = 5).
Blinding	The investigators were blinded to group allocation during data collection and analysis.

Reporting for specific materials, systems and methods

We require information from authors about some types of materials, experimental systems and methods used in many studies. Here, indicate whether each material, system or method listed is relevant to your study. If you are not sure if a list item applies to your research, read the appropriate section before selecting a response.

Materials & experimental systems

n/a	Involved in the study	
☐	☒	Antibodies
☐	☒	Eukaryotic cell lines
☒	☐	Palaeontology and archaeology
☐	☒	Animals and other organisms
☒	☐	Human research participants
☒	☐	Clinical data
☒	☐	Dual use research of concern

Methods

n/a	Involved in the study	
☒	☐	ChIP-seq
☐	☒	Flow cytometry
☒	☐	MRI-based neuroimaging

Antibodies

Antibodies used	PD-L1 antibody is provided by Bioxcell company(CAT.NO. BE0101-50MG,10F.9G2), FITC-conjugated anti-mouse CD8+ provided by Thermo (CAT. NO.06112 -60-100), PE-conjugated anti-Granzyme B provided by Thermo (CAT.NO. ab 225471), Fixable Viability Dye eFluor 450 (CAT. NO. 25-1971-82), Anti-Mouse CD16/32 provided by Biogems (CAT.NO. 20M 1218081221).
Validation	1.Stathopoulou, C., et al. (2018). "PD-1 inhibitory receptor downregulates asparaginyl endopeptidase and maintains Foxp 3 transcription factor stability in induced regulatory T Cells." Immunity 49(2): 247-263.<br>2.Aloulou, M., et al. (2016). "Follicular regulatory T cells can be specific for the immunizing antigen and derive from naive T cells." Nat Commun 7: 10579.

Eukaryotic cell lines

Policy information about cell lines

Cell line source(s)	4T1 cell line was obtained from ATCC, and the ATCC number are ATCC® CRL-2539™. DC 2.4 cell line was obtained from the Chinese Academy of Sciences cell library.
Authentication	Cell lines were authenticated by ATCC and the Chinese Academy of Sciences cell library.
Mycoplasma contamination	The cell lines were not tested for mycoplasma contamination.
Commonly misidentified lines (See ICLAC register)	No misidentified cell lines were found in this study.

Animals and other organisms

Policy information about studies involving animals; ARRIVE guidelines recommended for reporting animal research

Laboratory animals	Four-to-five-week-old female Balb/C mice were purchased from Shanghai Laboratory Animal Center (SLAC, shanghai, China), and were bred in a sterilized, specific pathogen-free (SPF) Lab of Tongji University.
Wild animals	The study was not involved in wild animals.
Field-collected samples	The study did not involve samples collected from the field.
Ethics oversight	Animal study protocols were approved by Tongji University Experimental Animal Center.

Note that full information on the approval of the study protocol must also be provided in the manuscript.

Flow Cytometry

Plots

Confirm that:

- [x] The axis labels state the marker and fluorochrome used (e.g. CD4-FITC).
- [x] The axis scales are clearly visible. Include numbers along axes only for bottom left plot of group (a 'group' is an analysis of identical markers).
- [x] All plots are contour plots with outliers or pseudocolor plots.
- [x] A numerical value for number of cells or percentage (with statistics) is provided.

Methodology

Sample preparation	All tissues were obtained from Balb/C mice in this study. The tissues inflated in digestion solution and incubated for 30 mins at 37 °C with continuous shaking every 5-8 min and gently grinded to acquire single-cell suspensions after passing the digested organs through a 70 µm strainer.
Instrument	Flow cytometry (BD FACS Aria II, USA) is used to collect the flow cytometry data.
Software	Flowjo(10) was used to analyze the flow cytometry data.
Cell population abundance	The amounts of cells in flow tube tested by flow cytometry (BD FACS Aria II, USA) are 5*10^5 /tube.
Gating strategy	The preliminary FSC-A/SSC-A gates of the starting cell population were 50K-150K/10^3-10^5. Cells were further stained by immunophenotyping antibodies with FITC-CD8, PE-Granzyme B. Subsequently, cells were stained with Fixable Viability Dye eFluor 450 and observed using BD flow cytometry. the cells that FITC-CD8, PE-Granzyme B and Dye eFluor 450 positive were cytotoxic T lymphocytes (CTLs).

- [x] Tick this box to confirm that a figure exemplifying the gating strategy is provided in the Supplementary Information.
